# Genomic and transcriptomic analyses of *Phytophthora cinnamomi* reveal complex genome architecture, expansion of pathogenicity factors, and host-dependent gene expression profiles

**DOI:** 10.3389/fmicb.2024.1341803

**Published:** 2024-08-15

**Authors:** Aidan C. Shands, Guangyuan Xu, Rodger J. Belisle, Shirin Seifbarghi, Natasha Jackson, Aureliano Bombarely, Liliana M. Cano, Patricia M. Manosalva

**Affiliations:** ^1^Department of Microbiology and Plant Pathology, University of California, Riverside, Riverside, CA, United States; ^2^Instituto de Biología Molecular y Celular de Plantas, Consejo Superior de Investigaciones Científicas-Universidad Politécnica de Valéncia, Valencia, Spain; ^3^Department of Plant Pathology, Indian River Research and Education Center (IRREC), Institute of Food and Agricultural Sciences (IFAS), University of Florida, Fort Pierce, FL, United States

**Keywords:** *Phytophthora*, oomycete, Phytophthora root rot, two-speed genome, effectors, cell wall-degrading enzymes, RXLRs, transcriptome analyses

## Abstract

*Phytophthora cinnamomi* is a hemibiotrophic oomycete causing Phytophthora root rot in over 5,000 plant species, threatening natural ecosystems, forestry, and agriculture. Genomic studies of *P. cinnamomi* are limited compared to other *Phytophthora* spp. despite the importance of this destructive and highly invasive pathogen. The genome of two genetically and phenotypically distinct *P. cinnamomi* isolates collected from avocado orchards in California were sequenced using PacBio and Illumina sequencing. Genome sizes were estimated by flow cytometry and assembled *de novo* to 140–141 Mb genomes with 21,111–21,402 gene models. Genome analyses revealed that both isolates exhibited complex heterozygous genomes fitting the two-speed genome model. The more virulent isolate encodes a larger secretome and more RXLR effectors when compared to the less virulent isolate. Transcriptome analysis after *P. cinnamomi* infection in *Arabidopsis thaliana, Nicotiana benthamiana*, and *Persea americana* de Mill (avocado) showed that this pathogen deploys common gene repertoires in all hosts and host-specific subsets, especially among effectors. Overall, our results suggested that clonal *P. cinnamomi* isolates employ similar strategies as other *Phytophthora* spp. to increase phenotypic diversity (e.g., polyploidization, gene duplications, and a bipartite genome architecture) to cope with environmental changes. Our study also provides insights into common and host-specific *P. cinnamomi* infection strategies and may serve as a method for narrowing and selecting key candidate effectors for functional studies to determine their contributions to plant resistance or susceptibility.

## 1 Introduction

*Phytophthora* species' overall economic damage to crops in the United States is estimated to be in the tens of billions of dollars, including the costs of control measures (Kamoun et al., 2015). The hemibiotrophic oomycete, *Phytophthora cinnamomi* Rands causes Phytophthora root rot (PRR) in over 5,000 plant species, threatening natural ecosystems, agriculture, nursery, and forestry industries (Cahill et al., 2008; Jung et al., 2013, 2018; Kamoun et al., 2015; Hardham and Blackman, 2018; Shakya et al., 2021). Avocado PRR, caused by *P. cinnamomi*, continues to be the major hindrance to avocado production worldwide (Ramírez-Gil et al., 2021). In 2022, California, which produced 88% of the domestic avocado crop (USDA NASS) (US Department of Agriculture: National Agricultural Statistics Service, 2022), remains challenged by this pathogen, affecting approximately 60–75% of the avocado growers and causing annual losses of $40 million (Coffey, 1987). Losses to this pathogen not only decrease crop yield and product value but also cause significant expenditures for control measures. Growers rely mostly on available resistant rootstocks and phosphonate-based fungicides for PRR management (Morse et al., 2016); however, pathogen isolates partially overcoming both practices have been detected in California (Belisle et al., 2019a,b). *Phytophthora cinnamomi* is heterothallic with two mating types, A1 and A2; however, only clonal populations of the A2 mating type have been found to affect avocado orchards worldwide (Dobrowolski et al., 2003; Pagliaccia et al., 2013; Shakya et al., 2021; Engelbrecht et al., 2022). Many *Phytophthora* spp., including *P. cinnamomi*, are known to exhibit genetic and phenotypic variabilities despite their clonality (Oudemans and Coffey, 1991; Linde et al., 1999; Dobrowolski et al., 2003; Eggers et al., 2012; Pagliaccia et al., 2013; Belisle et al., 2019a,b). Pagliaccia et al. (2013) reported a significant population structure among *P. cinnamomi* affecting California avocado orchards. Two genetically distinct clades of A2 mating type isolates, clade I and clade II, were identified, with the latter clade only found in Southern California growing regions. Belisle et al. (2019b) reported that the clade II isolates exhibited slower growth, less sensitivity to higher temperatures and potassium phosphite fungicide, and higher virulence in avocados.

The reduced cost of next-generation sequencing technologies in the last decade facilitated the development of several *Phytophthora* spp. genome assemblies (Shi et al., 2021; Wang et al., 2021; Carleson et al., 2022; Cox et al., 2022; Matson et al., 2022). Prior to 2020, five *P. cinnamomi* genome assemblies were publicly available; however, these assemblies were largely fragmented due to the use of second-generation sequencing technologies (Studholme et al., 2016; Longmuir et al., 2017; https://genome.jgi.doe.gov/Phyci1). Recently, Engelbrecht et al. (2021) reported a high-quality *P. cinnamomi* genome assembly using Oxford Nanopore and Illumina sequencing platforms. The availability of these genomic resources helped to detect hybrids (Hamelin et al., 2022), develop markers for population studies (Fan et al., 2022), and gain insights into the infection strategies, evolution, and adaptation of these destructive oomycete pathogens (Engelbrecht et al., 2021; Carleson et al., 2022; Cox et al., 2022; Midgley et al., 2024).

Several studies argue that effector proteins are major contributors to *Phytophthora*'s pathogenicity, virulence, and adaptation (Birch et al., 2009; Bos et al., 2010; Dong et al., 2011; Hardham and Blackman, 2018; Dong and Ma, 2021). Pathogen effectors are proteins that exhibit a wide range of avirulence and virulence functions and are secreted either to the host apoplast or translocated into the cytoplasm and intracellular compartments (Oliva et al., 2015; Franceschetti et al., 2017; Armitage et al., 2018). Apoplastic effectors, such as elicitins and necrosis-inducing proteins (NIPs), have been shown to elicit immune responses, including cell death and necrosis, to aid in infection, respectively (Osman et al., 2001; Rodrigues et al., 2006; Hardham and Blackman, 2018). Cytoplasmic effectors, such as RXLRs, have diverse roles, including interfering with host defense mechanisms or suppressing the function of other effectors (Bos et al., 2010; Kelley et al., 2010; Bozkurt et al., 2011, 2012). Some *Phytophthora* spp. tend to harbor a large proportion of RXLRs in expanded gene-sparse repeat-rich regions, leading researchers to hypothesize that these regions could promote genome plasticity and increase the genetic variation of effectors for evolutionary adaptation (Haas et al., 2009; Raffaele et al., 2010; Dong et al., 2015). Additional apoplastic effectors include cell wall-degrading enzymes (CWDE), which are secreted by pathogens including *Phytophthora* spp. to aid infection and have been shown to be key pathogenicity and virulence factors for phytopathogens (Blackman et al., 2014; Kubicek et al., 2014). Other effectors, such as carbonic anhydrases (CAs), have been implicated as virulence factors in *P. infestans* and hypothesized to be involved in inducing or enhancing host cell death responses during the necrotrophic stage (Raffaele et al., 2010). Several transcriptomic and functional studies of *Phytophthora* effectors at pre-infection stages and during *in planta* infection revealed their importance during the infection process and plant immunity (Evangelisti et al., 2017; Reitmann et al., 2017; Gumtow et al., 2018; Pettongkhao et al., 2020; Joubert et al., 2021; Luo et al., 2021; Midgley et al., 2024).

Despite the recent genomic resources generated for *P. cinnamomi*, there remain many gaps regarding mechanisms for generating genetic diversity, understanding the genomic architecture, elucidating host-infection strategies, and comparing the genomes of phenotypically distinct clonal isolates. Our research aims to produce additional high-quality *P. cinnamomi* genome assemblies of two phenotypically distinct isolates to uncover genomic evidence that could explain their variability. Transcriptomic analyses of *P. cinnamomi* infection in multiple hosts were performed to reveal common and host-specific infection strategies to better understand *P. cinnamomi* host adaptation and pathogenicity. This study generated novel genomic and transcriptomic resources that can aid in the development of control methods, such as the design of DNA- or protein-based diagnostic tools. Moreover, this study will aid in the selection of candidate genes for functional studies in *P. cinnamomi* to better understand their contributions to pathogenicity or virulence and identify their corresponding plant susceptibility factors for the development of *P. cinnamomi*-resistant plants.

## 2 Material and methods

### 2.1 Pathogen isolates

*Phytophthora cinnamomi* isolates Pc2113 (A2 clade I) and Pc2109 (A2 clade II) were obtained from the Manosalva's laboratory pathogen collection, the CBS 144.22 isolate from the World Phytophthora Genetic Resource Collection (WPC) (University of California, Riverside), and the *P. infestans* isolate 1306-C (Pi1306-C) from Dr. Howard Judelson, UCR (Pan et al., 2018). *Phytophthora* isolates in this study were maintained as water agar plugs (Boesewinkel, 1976) and stored at 22°C.

### 2.2 Plant material and growth conditions

*Raphanus sativus* cv. Saxa (radish), *Solanum lycopersicum* cv. M82 (tomato), and *Nicotiana benthamiana* seeds were germinated and grown under a 16-h light/8-h dark photoperiod at 22°C with 70% relative humidity. Two-week-old tomato seedlings were transferred to a greenhouse with an average air temperature between 25 and 28°C and an average humidity of 56%. Clonal Dusa^®^ avocado rootstock liners (Westfalia Technological Services, Tzaneen, South Africa) were obtained from Brokaw Nursery LLC (Santa Paula, Ventura, CA). The 6-month-old liners were removed from their bags and transplanted into pots after the nurse seed was removed. Plants were grown in a greenhouse under the conditions described above. *Arabidopsis thaliana* ecotype Landsberg erecta (L*er*-0) were germinated and grown in a Conviron Adaptis A1000 growth chamber (Controlled Environments Limited, Winnipeg, Manitoba, Canada) at 23°C, 60% relative humidity, with a 16-h light/8-h dark photoperiod.

### 2.3 DNA extraction

All *Phytophthora cinnamomi* isolates used in this study were grown in a liquid Plich media (Van der Lee et al., 1997) for 5–7 days at 22°C in the dark. The mycelia were harvested, washed with sterile Milli-Q water (MilliporeSigma, Burlington, MA, USA), flash-frozen in liquid nitrogen, and ground to a fine powder using a mortar and pestle. Approximately 100 mg of mycelia were transferred to microcentrifuge tubes and stored at −80°C until needed. Pc2113 and Pc2109 high-molecular-weight genomic DNA (gDNA) was extracted using the OmniPrep™ for Plants kit (G-Biosciences, St. Louis, MO, USA) according to the manufacturer's protocol, except for an additional RNAse treatment which involved adding 1.5 μl of LongLife™ RNAse (G-Biosciences, St. Louis, MO, USA) to the DNA solution and incubation at 37°C for 30 min. CBS 144.22 gDNA was extracted using the DNeasy Plant Mini kit (Qiagen, Germantown, MD) according to the manufacturer's instructions.

### 2.4 Genome sequencing

Pc2109 and Pc2113 gDNA were subjected to two independent Pacific Biosciences (PacBio) RS II and Sequel (PacBio, Menlo Park, CA) library preparations and sequencing runs at both Icahn Medical Institute at Mount Sinai (New York, NY) and Novogene Corporation (Sacramento, CA), respectively. Paired-end gDNA libraries with an insert size of 350 bp were generated for each isolate and sequenced using the Illumina NovaSeq 6000 PE150 platform (Illumina, San Diego, CA) by Novogene Corporation.

### 2.5 K-mer and flow cytometry analyses for genome size estimation

Flow cytometry (FCM) was conducted before genome assembly to determine the nuclear content. *Phytophthora* isolates were grown in 10% clarified V8 broth (cV8) (Belisle et al., 2019b) under the conditions indicated above for 3 days. Mycelium harvest, nuclei extraction, and staining were performed using the Cystain^®^ PI Absolute P kit (Sysmex Co., Kobe, Hyogo, Japan) as described by Bertier et al. (2013). *Raphanus sativus* cv. Saxa and/or *Solanum lycopersicum* cv. M82 leaf tissue served as reference standards. Approximately 10,000 nuclei per sample were measured in triplicate using MoFlow^®^ Astrios EQ Cell Sorter (Beckman Coulter, Indianapolis, IN) equipped with a 200 mW laser (488 nm). The data was analyzed using FlowJo™ software v10.7 (Tree Star Inc., Ashland, OR), and the genome size estimations were calculated as described by Doležel et al. (2007). The diploid *P. infestans* isolate Pi1306-C nuclear content was estimated and served as an FCM control. Genome size estimation was also performed using k-mer counting. K-mer histograms were generated with trimmed Illumina reads using Jellyfish (v2.3.0) (k-mer range 17–99 increments of 7) (Marçais and Kingsford, 2011), and the respective k-mer histograms served as the input for GenomeScope (accessed 20 November 2018) (Vurture et al., 2017) profiling.

### 2.6 *De novo* genome assembly, genome completeness, and whole genome alignments

PacBio subreads were combined and used for each *de novo* genome assembly using Canu v1.7.1 (Koren et al., 2017). The resulting assemblies were corrected with PacBio reads iteratively three times using Racon v1.3.2 (Vaser et al., 2017). The Illumina reads were filtered using Trimmomatic v0.36 (Bolger et al., 2014) and used to polish the Racon-corrected assemblies iteratively three times using Pilon v1.22 (Walker et al., 2014). Purge Haplotigs (Roach et al., 2018) were used to obtain a haploid genome assembly size that was consistent with our FCM estimations. Benchmark Universal Single-Copy Orthologs (BUSCO) v5.7.1 (Manni et al., 2021) using the Stramenopile database (orthoDB v.10) (Kriventseva et al., 2019) was used to assess assembly completeness. Assembly statistics were generated using QUAST v4.6.3 (Gurevich et al., 2013), and their quality and heterozygosity were assessed with KAT v2.3.4 (Mapleson et al., 2017). Pc2113 and Pc2109 whole genome alignments were performed using Nucmer (MUMmer v4.0), and differences were assessed using DNAdiff (Marçais et al., 2018). Dot plots were generated using Minimap2 v2.10 (Li, 2018) and visualized using D-GENIES v1.3.1 (Cabanettes and Klopp, 2018). Cross-read mapping was performed with Pc2113 and Pc2109 raw Illumina reads to the GKB4 *P. cinnamomi* reference genome (NCBI accession number PRJNA675400) using BWA-mem v0.7.17 (Li, 2013), and mapping percentage was acquired using Samtools v1.18 flagstats (Li et al., 2009).

### 2.7 Genome annotation

First, repeat annotation and transposable element identification were performed using RepeatModeler 2 v2.0.1 (http://www.repeatmasker.org) for both assemblies. Assemblies for Pc2113 and Pc2109 were merged and used to build the database for each isolate. The respective database was used to annotate repeats, and a soft mask was performed for each genome using RepeatMasker v4.0.9 (http://www.repeatmasker.org) before conducting the gene annotation process using RNA-seq data as supporting gene evidence. The RNA sequences were trimmed and filtered using fastq-mcf v1.05 (Aronesty, 2011) (-q 30 -l 50), and alignments were performed with STAR v2.7.5a (Dobin et al., 2013) and assembled using StringTie v2.1.5 (Pertea et al., 2015). GFF utilities (Pertea and Pertea, 2020) were used to convert the GTF from StringTie to GFF to extract mRNA sequences. Peptide prediction was made with TransDecoder v5.5.0 (https://github.com/TransDecoder/), DIAMOND v0.9.24 (Buchfink et al., 2014), and HMMER3 (Eddy, 2009) (hmmscan v3.1b2). BRAKER v2.1.5 (Hoff et al., 2019) pipeline, GeneMark v4.61 (Stanke et al., 2006), and Augustus v3.3.3 (Stanke and Morgenstern, 2005) were employed to predict genes. Gene annotation was also conducted using MAKER pipeline v3.01.02, for which the *ab initio* gene prediction program SNAP v2006-07-28 (Korf, 2004) was trained with the mRNA sequences and peptides described above. The final round of MAKER was completed using the proteins from TransDecoder, including *P. infestans* v2 (NCBI accession number PRJNA17665), *P. ramorum* v1 (JGI Project ID 16273), *P. sojae* (JGI Project ID 16274), *P. parasitica* v2 (NCBI accession number PRJNA73155), *P. capsici* v11 (JGI Project ID 16272), *P. cinnamomi* v1 (JGI Project ID 1003774), and *P. nicotianae* v.1 (NCBI accession number PRJNA294216). BUSCO v4.1.4 (Simão et al., 2015) was used to assess the completeness of the annotations (BRAKER & MAKER) as described above, and MAKER and BRAKER annotation comparison was performed using Mikado v2.0rc2 (Venturini et al., 2018). BRAKER was chosen as the “base” annotations, and the MAKER annotations served as the “donor”. Unique genes from the maker were joined with the BRAKER annotations and the GFF file was sorted (https://github.com/billzt/gff3sort, v2017). DIAMOND was conducted for functional annotations using the SwissProt and TREMBL datasets (accessed on 3 September 2019). Descriptions were added to the annotations using AHRD v3.3.3 (https://github.com/groupschoof/AHRD).

### 2.8 Ploidy analysis

*Phytophthora cinnamomi* and *P. infestans* ploidies were assessed using nQuire (Weiß et al., 2018) and the *vcfR* package (Knaus and Grünwald, 2017) in R v4.1.2 (R Core Team, 2010). The Illumina reads were trimmed, filtered using Fastq-mcf v1.1.2-537 (Aronesty, 2011), and the reads were mapped to their corresponding reference genomes as indicated above. The respective BAM files were processed using Samtools and assessed with Qualimap v2.2.1 (García-Alcalde et al., 2012). Single-nucleotide polymorphisms (SNPs) were called from the processed BAM files using FreeBayes v1.2.0 (Garrison and Marth, 2012). The respective variant call format file (VCF) was filtered with VCFtools v0.1.16-18 and imported into R for allele balance analysis following the methods described in Knaus and Grünwald (2018). The *P. infestans* isolate 1306-C was subjected to the same analysis as a diploid control using the T30-4 reference genome (NCBI accession number ASM14294v1).

### 2.9 Gene orthology analysis

*Phytophthora infestans* (NCBI accession number PRJNA17665), *P. ramorum* (NCBI accession number PRJNA12571), *P. sojae* (JGI Project ID 16274), *P. fragariae* (NCBI accession number PRJNA396163), *P. parasitica* (NCBI accession number PRJNA73155), and *P. capsici* (NCBI accession number PRJNA481983) proteomes were used to conduct orthology analysis using Orthofinder v2.5.2 (Emms and Kelly, 2019). Orthogroup intersections were visualized using *UpsetR* v1.4.0 (Conway et al., 2017) and extracted using *ComplexHeatmap* v2.6.2 (Gu et al., 2016) for downstream analysis. To identify proteins only present in *P. cinnamomi*, a tBLASTn v2.9.0+ analysis was performed (Altschul et al., 1997). tBLASTn results were filtered based on ≥80% identity and ≥80% of the query alignment length. The respective proteins were then subjected to Exonerate v2.4.0 (Slater and Birney, 2005) and filtered based on ≥80% identity and similarity. In addition, separate orthology analyses using GKB4 (Engelbrecht et al., 2021), Pc2113 and Pc2109 proteomes, predicted carbohydrate-active enzymes (CAZymes), and RXLRs were performed.

### 2.10 Two-speed genome analysis, secretome analysis, and effector predictions

To determine the genome architecture for each *P. cinnamomi* isolate, two-dimensional gene binning based on their flanking intergenic regions' (FIRs) length was performed using methods adopted by Saunders et al. (2014) and visualized using the density-Mapr R script (https://github.com/Adamtaranto/density-Mapr). Gene-dense regions (GDRs) and gene-sparse regions (GSRs) of the genomes were delimited using methods described by Raffaele et al. (2010) and Rojas-Estevez et al. (2020) (Supplementary Method S1). The proteome for each *P. cinnamomi* isolate was scanned for signal peptide (SP) presence using SignalP (v5.0) (Armenteros et al., 2019). The resulting proteins containing SP sequence were subjected to TMHMM (v.2.0) analyses (Möller et al., 2001) to identify transmembrane domains (TMDs). TMD-containing proteins were removed from the secretome datasets. Secretomes were subjected to EffectorP v3.0 (Sperschneider and Dodds, 2022) to predict apoplastic, cytoplasmic, and dual-localized effectors. In addition, RXLR effectors were also predicted using regular expression (REGEX) searches following methods described by Win et al. (2007) and Boutemy et al. (2011) (Supplementary Method S2). Multiple sequence alignments of the predicted Pc2113 and Pc2109 RXLRs, 61 GKB4 RXLRs, and 41 RXLRs from other *Phytophthora* spp. and *Hyaloperonospora arabidopsis* described in Joubert et al. (2021) were performed with MUSCLE v3.8.425 (Edgar, 2004) using default settings. A maximum likelihood phylogenetic tree was generated using IQ-Tree2 v2.1.3 (Minh et al., 2020) with the VT+R7 model determined by ModelFinder (Kalyaanamoorthy et al., 2017). The phylogenetic tree was visualized using the *ggtree* v3.2.1 (Yu et al., 2017) and *ComplexHeatmap* R packages. CAZymes were identified using the dbCAN2 web server (Yin et al., 2012; Zhang et al., 2018) using the CAZyme database (Cantarel et al., 2009), as described by Engelbrecht et al. (2021). Proteins were considered CAZyme if they were positive for two or more programs (HMMER, Hotpep & Diamond). Proteins were putatively classified as cell wall-degrading enzymes (CWDEs) if they contained polysaccharide lyase (PL), carbohydrate esterase (CE), glycoside hydrolase (GH), auxiliary activity (AA), or carbohydrate-binding module (CBM) modules, which are associated with plant cell wall degradation (Blackman et al., 2014). Necrosis-inducing proteins (NIPs), carbonic anhydrases (CAs), crinkling and necrosis (CRNs), elicitins, and elicitin-like proteins were identified from the functional annotations generated in this study.

### 2.11 Pathogen inoculation and RNA-seq analyses

Leaves of 5-week-old *N. benthamiana*, 9-month-old Dusa^®^ avocado, and three-and-a-half-week-old *A. thaliana* L*er*-0 were used for Pc2113 detached leaf inoculations as described in Belisle et al. (2019b). At 16- and 24-h post-inoculation (hpi), three infected leaf discs were harvested from three individual plants as one biological replicate per time point. Three biological replicates were used for this RNA-seq experiment, apart from *A. thaliana* samples at 90 hpi, which consisted only of two biological replicates. Leaf tissue was frozen in liquid nitrogen, ground to a fine powder using a mortar and a pestle, and stored at −80°C. Pc2113 mycelia bearing sporangia were harvested, vacuum-filtered, ground to a fine powder in liquid nitrogen with a mortar and pestle, and stored at −80°C. Pc2113 zoospore suspension (2 × 10^5^ zoospore/ml) was centrifuged at 362 × *g* for 5 min and the resulting pellet was resuspended in 1 ml of sterile water, ground in liquid nitrogen using a mortar and a pestle, and stored at −80°C. Mycelia-bearing sporangia and zoospore samples were harvested in three biological replicates. Total plant and pathogen RNA were extracted using the RNeasy Plant Mini Kit (Qiagen, Germantown, MD, USA) according to the manufacturer's instructions, followed by DNAse treatment with the Invitrogen TURBO DNA-free™ Kit (Thermo Fisher Scientific, Waltham, MA). RNA cleanup and purification were performed using the Zymo RNA Clean and Concentrator Kit (Zymo Research Corp., Irvine, CA). RNA pellets were resuspended in nuclease-free water, and the total RNA concentration and absorbance 260/280 ratio, indicative of RNA purity, were measured using a NanoDrop (Thermo Fisher Scientific, Waltham, MA). To confirm successful infection and pathogen accumulation before submitting samples for RNA sequencing, *P. cinnamomi* biomass at the respective time points post-inoculation was calculated using TaqMan real-time PCR as described by Belisle et al. (2019b). Agilent 2100 Bioanalyzer (Agilent Technologies, Santa Clara, CA) using the Plant RNA Nano Assay was used to assess the RNA quality and purity before sending samples to Novogene for library preparation and 250–300 bp paired-end sequencing using the Illumina HiSeq2 platform. RNA sequencing reads were mapped to the Pc2113 assembly with HISAT2 v2.1.0 (Kim et al., 2019) using default settings. Samtools v1.12 was used for alignment mapping file processing, and reads were counted with HTseq-count v0.11.1 (Anders et al., 2015). Differential gene expression analysis was conducted using the R package *DEseq2* v1.30.1 (Love et al., 2014). Genes with < 10 counts across the rows of the matrix were removed, and differential expression analysis was performed using the *DEseq* function. Comparisons within DEseq2 were performed by comparing the *in planta* timepoints to the Pc2113 mycelium (bearing sporangia) using the *contrast* function with the following settings: *alpha* = 0.05 and *lfcThreshold* = 1. Genes were characterized as differentially expressed (DE) if the false discovery rate adjusted *p* values (Benjamini and Hochberg, 1995) were of < 0.05 and had a |log-fold change (LFC)| of ≥ 2. Genes with an LFC of ≥ 2 were considered upregulated, and genes with an LFC of ≤ -2 were considered downregulated. The same method was applied to compare Pc2113 sporulating mycelium and Pc2113 zoospore samples to identify DE genes in the zoospore sample. Gene expression in terms of transcript per million (TPM) was generated using the same bam files described previously using StringTie v2.2.1 (Pertea et al., 2015). The transcript TPM values were extracted from the output GTF files and averaged across each of the sample replicates at each timepoint, respectively. Genes were considered expressed if the TPM > 5. The respective TPM values were log_2_ transformed for visualization using *ComplexHeatmap* v.2.6.2 in R.

### 2.12 qPCR analyses

qRT-PCR was used to validate expression patterns identified by the RNA-seq data with the primers in Supplementary Table S1. qRT-PCR analysis of three highly upregulated *P. cinnamomi* effector genes was performed to validate RNA-seq results. Detached leaf inoculations with Pc2113 in *N. benthamiana* were conducted as described above. Leaf discs were harvested for three replicate samples, with three plants per replicate at each time point. cDNA synthesis was performed using the Invitrogen Superscript III Kit (Thermo Fisher Scientific, Waltham, MA) following the manufacturer's protocol. Each 10-μl reaction included 4 μl of a cDNA (1:20 dilution), 5 μl of iQ SYBR Green Supermix (Bio-Rad Laboratories, Inc., Hercules, CA), and 0.5 μM of each forward and reverse qRT-PCR primers. Reactions were amplified using a CFX384 Touch Bio-Rad Real-Time PCR system (Bio-Rad Laboratories) under the following conditions: 95°C for 3 min (initial denaturation), followed by 40 cycles of denaturation at 95°C for 10 s, annealing/extension at 60°C for 30 s, and a melt curve of 5 s at 65–95°C. Gene expression levels were calculated using the 2^−Δ*ΔCt*^ (Ct = cycle threshold) comparative method (Livak and Schmittgen, 2001). The results were reported as the mean ± standard error of three biological replications and three technical replications. Pearson's correlation test was used to evaluate significance. All primers in this study were designed using Primer3 v0.4.0 software. The three candidate gene primer pairs used (sequences not shown) were Pc2113T1C00002279g0000010.1 (RXLR052), Pc2113T1C00002373g0000080.1 (Elicitin2373), and Pc2113T1C00002266g0000100.1 (RXLR047) (Supplementary Table S1). *Phytophthora cinnamomi* 40s ribosomal gene Ws21 (86101) was used as an endogenous reference gene for normalization. Primer efficiency for each pair was calculated via the slope of a standard curve constructed by the amplification of a 10-fold dilution series of DNA [covering five dilution points, 1–10,000 picograms (pg)]. All primer sets were between 95% and 99% efficient. This experiment was conducted two times.

## 3 Results

### 3.1 *Phytophthora cinnamomi* isolates collected from avocado orchards in California exhibited larger genome sizes than previously reported

Raw Illumina reads post-filtering resulted in a total of ~156 and ~130 million reads, representing 23.1 and 19.1 GB of filtered data for Pc2113 and Pc2109, respectively (Supplementary Table S2). Genome size estimations by GenomeScope yielded a size range from 144.5 Mb (17-mer) to 178.6 Mb (99-mer) for Pc2113 and from 140.4 Mb (17-mer) to 183.6 Mb (86-mer) for isolate Pc2109 (data not shown). Recent studies reported the predominance of triploidy in *P. cinnamomi* populations collected from avocado orchards in South Africa (Engelbrecht et al., 2021, 2022). Considering that Pc2113 and Pc2109 genome size estimations were close to the South African triploid *P. cinnamomi* isolate GKB4 (Engelbrecht et al., 2021), we used a triploid model to calculate the haploid genome size of the *P. cinnamomi* isolates by flow cytometry. Consistent with Van Poucke et al. (2021), all the *P. cinnamomi* isolates from this study exhibited similar DNA content, with an average ranging from 0.41 to 0.43 pg (Supplementary Figure S1). The use of *R. sativus* (2C = 1.09 pg) (Arumuganathan and Earle, 1991) as the reference standard resulted in a slight overlap of the *P. cinnamomi* G2_P_ and *R. sativus* G1_R_ nuclear populations (Supplementary Figures S1A–C). To resolve the overlap, *S. lycopersicum* (2C = 2.01 pg) (Marie and Brown, 1993) was substituted as a reference standard (Supplementary Figure S1D). No significant differences (*p* = 0.7187, Kruskal–Wallis rank sum test) were observed in the estimated DNA content (pg) for the Pc2113 isolate using either reference standard (Supplementary Figure S1E). As expected, we obtained an estimated genome size of 0.54 pg for *P. infestans* isolate Pi1306-C (Catal et al., 2010; Wang et al., 2016) (Supplementary Figure S1E). These results indicate a larger genome size for California *P. cinnamomi* isolates compared to the other publicly available *P. cinnamomi* genome assemblies, and these estimations were used to guide the respective *de novo* genome assemblies.

Post-filtering of the PacBio reads resulted in approximately 1.8 million subreads for each isolate, representing ~20 Gb and ~1 Gb for Pc2113 and Pc2109, respectively (Supplementary Table S2). The initial raw Canu assemblies yielded 259.9 Mb and 232.9 Mb for Pc2113 and Pc2109, respectively. Three iterations of Racon followed by three iterations of Pilon increased the quality of the assemblies but resulted in larger genome sizes than those estimated by the K-mer and FCM analyses. Purge Haplotigs successfully reduced the number of heterozygous contigs observed within the assemblies to match the FCM estimations under triploidy. The resulting assemblies representing the haploid state yielded a length of 140.7 Mb and 141 Mb for the Pc2113 and Pc2109 genomes, respectively, in 735 and 897 contigs with N50 values ranging from 289 to 255 kb. BUSCO analyses indicated a completeness of 100% for each isolate, and 77–86% were single BUSCOs with duplications of 23% and 14% for Pc2113 and Pc2109, respectively. Genome annotation for Pc2113 and Pc2109 yielded 21,402 and 21,111 genes, respectively. In addition, we found 375 and 399 alternatively spliced variants and a total of 21,777 and 21,510 proteins for Pc2113 and Pc2109, respectively. The secretome of each isolate is comprised of 1,400 and 1,342 proteins for Pc2109 and Pc2113, respectively (Table 1).

**Table 1:** Genome assembly and annotation characteristics for each *P. cinnamomi* isolate.

	**Pc2113**	**Pc2109**
Number of contigs	735	897
Largest contig (Mb)	1.9	1.77
Assembly size (Mb)	140.7	141
GC (%)	54.37	54.26
N50	289 Kb	255 Kb
Complete BUSCOs (%)	100	100
Single (%)	77	86
Duplicated (%)	23	14
Fragmented (%)	0	0
Missing (%)	0	0
Number of genes	21,402	21,111
Number of alternative spliced variants	375	399
Number of total encoded proteins	21,777	21,510
Average gene length	1,378 bp	1,417 bp
Number of secreted proteins	1,342	1,400

### 3.2 High synteny, sequence similarity, and complexity were found between the triploid assemblies of two phenotypically distinct *Phytophthora cinnamomi* isolates from California

The ploidy of *P. cinnamomi* isolates Pc2113, Pc2109, and CBS 144.22 was confirmed as triploid based on two different approaches. *Phytophthora cinnamomi* allele balance histograms generated from vcfR displayed a binomial distribution with two dominant peaks at 1/3 and 2/3 typical for triploid isolates (Supplementary Figures S2A–C). As expected, the diploid Pi1306-C control isolate showed an allele balance histogram displaying a dominant peak at $\frac{1}{2}$, indicative of diploidy (Supplementary Figure S2D). nQuire ploidy models for *P. cinnamomi* isolates generated the lowest delta log-likelihood values for the triploid model, while the lowest value for *P. infestans* was for the diploid model (Supplementary Figure S2E).

**Figure 1:**
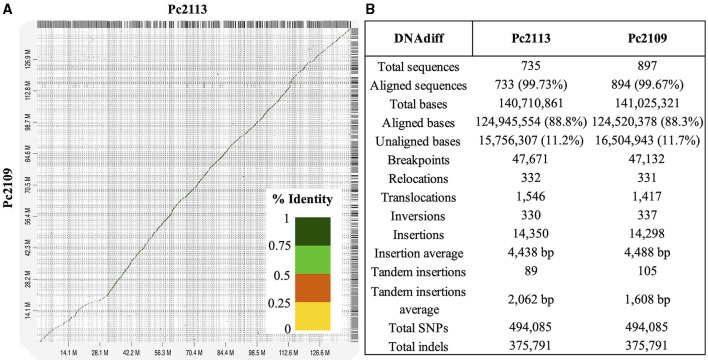
Whole-genome comparison of Pc2113 and Pc2109 genome assemblies. **(A)** Whole-genome alignment visualized as a dot plot with noise removed demonstrates high sequence similarity between the two genomes. Regions of homology are indicated as diagonal lines or dots color-coded by the percentage of sequence identity as indicated in the color bar ranging from 0 to 1 (100%). **(B)** DNAdiff summary statistics of the whole-genome alignment between Pc2113 and Pc2109.

**Figure 2:**
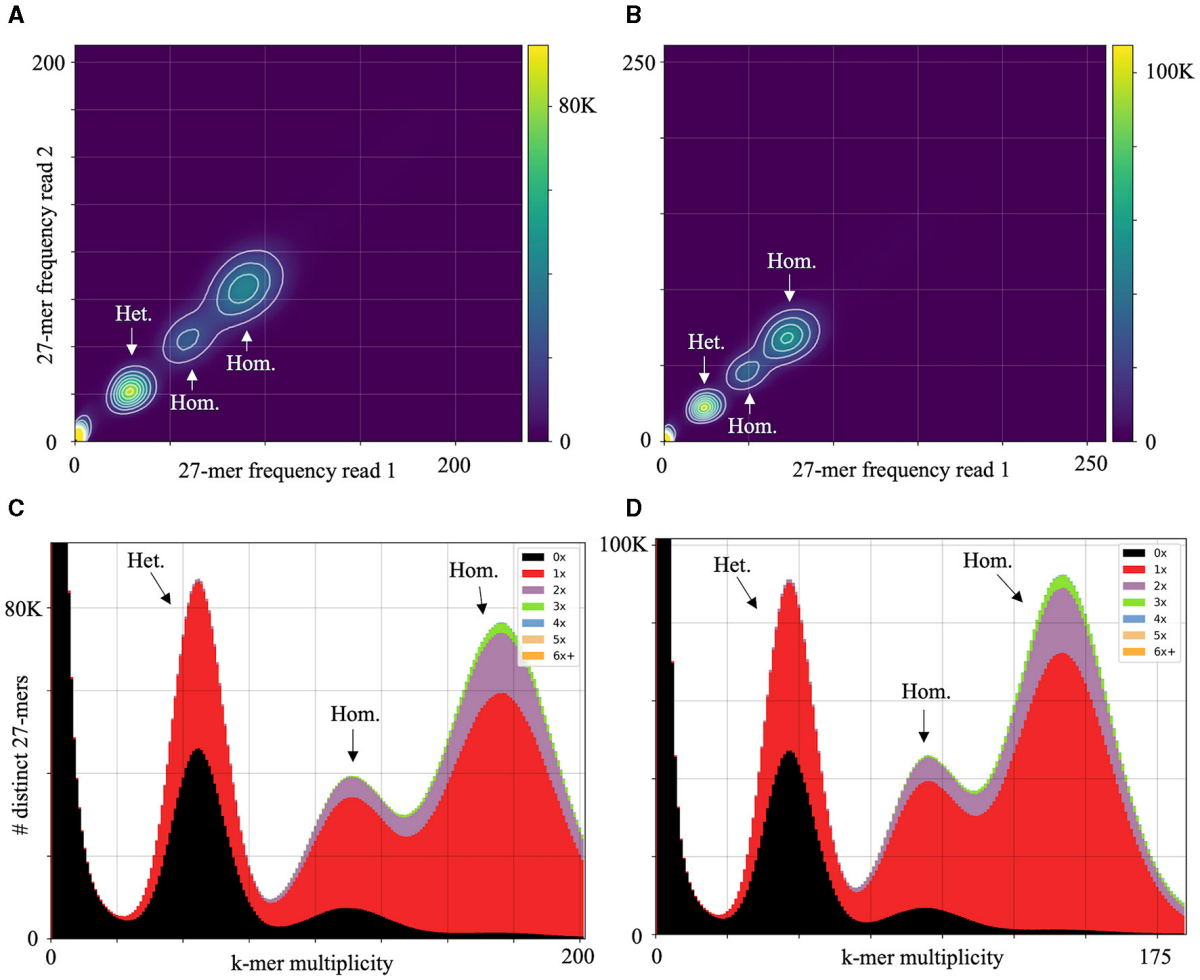
Genome complexity of Pc2113 and Pc2109 revealed by KAT k-mer analysis. KAT density plots display the 27-mer multiplicity of read 1 (*x*-axis) against read 2 (*y*-axis) for Pc2113 **(A)** and Pc2109 **(B)**. The bottom corner of the plots indicates low-frequency 27-mers due to sequencing errors or contamination. KAT k-mer spectra-cn plots displaying distinct 27-mers from Pc2113 **(C)** and Pc2109 **(D)**. Red represents the k-mers within the assembly, black represents k-mers present in the read set but not in the assembly, and the other colors represent k-mers that exhibited different levels of duplications within the assemblies. Each isolate displays three distinct regions indicated by arrows and labeled as homozygous (Hom.) and heterozygous (Het.).

High macrosynteny and sequence identity (>75%) were observed in the dot plot between Pc2109 and Pc2113, and each assembly with GKB4 (Figure 1A, Supplementary Figures S3A, B). Cross-read mapping of the California *P. cinnamomi* Illumina reads to the GKB4 assembly yielded a mapping percentage of >97%, supporting the observations in the respective dot plots. Pc2113 and Pc2109 genome comparison using DNAdiff also showed that 99.7% of their total contigs aligned with ~88% of sequence identity. No major differences were detected regarding breakpoints between both genomes; however, Pc2113 showed 539 more events than Pc2109, including translocations and insertions. Interestingly, DNAdiff revealed that the Pc2109 genome exhibited more tandem insertions (105) than Pc2113 (89), with an average size of 1.6 kb (Figure 1B). KAT k-mer density plots were generated to assess genome complexity. Both *P. cinnamomi* isolates exhibited similar distributions, with a strong heterozygous k-mer signal at a quarter coverage and two homozygous signals, one at half coverage (weak signal) and another at full coverage (strong signal) (Figures 2A, B). KAT spectra-cn plots were used to investigate the k-mer frequencies in common between the assemblies of Pc2113 and Pc2109 and their respective read sets. Consistent with the KAT density plots, each isolate displayed three distinct peaks (one heterozygous and two homozygous) (Figures 2C, D). The first homozygous peak is well represented in the respective assemblies, with a small portion of the read set (black) not captured in the assembly (red), while the second homozygous peak is almost completely represented in the assembly. In addition, both homozygous peaks from each isolate exhibited different levels of k-mer duplications (2 × and 3 × ) (Figures 2C, D). Together, these results indicated completeness and high genome complexity, with different levels of duplications supporting polyploidy and/or aneuploidy events.

### 3.3 California *P. cinnamomi* isolates exhibited private orthogroups

**Figure 3:**
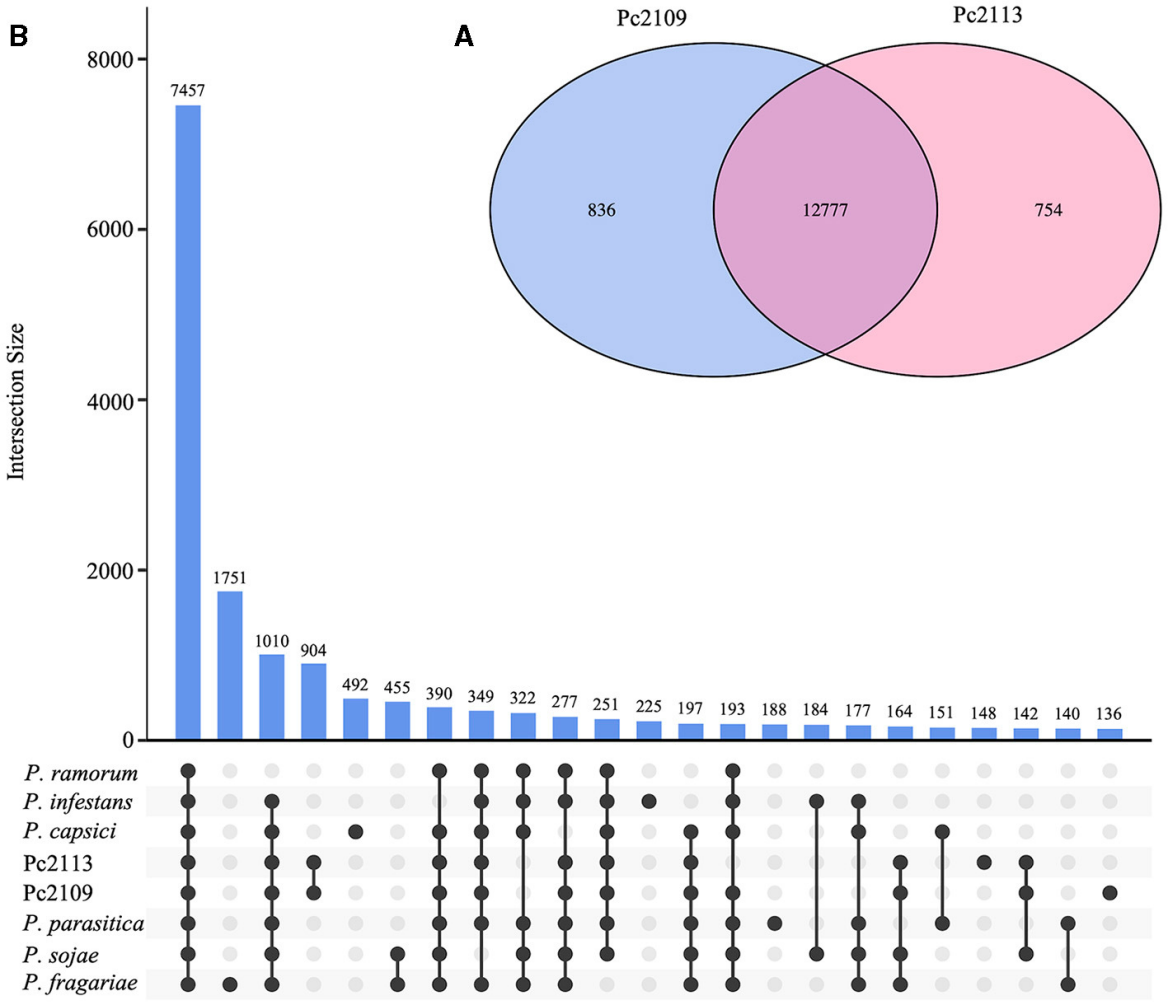
Orthology analysis of Pc2113, Pc2109, and six *Phytophthora* spp. proteomes. **(A)** Venn diagram showing the number of orthogroups shared between Pc2113 and Pc2109. **(B)** Upset plot displaying the number of orthogroups shared between the different *Phytophthora* spp. sets. Black dots on the *x*-axis represent the presence/absence of orthogroups in the specific set. Intersections are defined by a line connecting the black points and the corresponding intersection size is displayed above as bars. Only 23 intersections are shown in this plot.

Orthology analysis revealed that Pc2113 and Pc2109 shared 12,777 orthogroups, representing 88% of their respective proteomes. In Pc2113, there were 754 orthogroups containing 773 proteins that were absent in Pc2109. Interestingly, Pc2109, the most virulent isolate, exhibited more orthogroups (*n* = 836) comprised of 929 proteins not shared with Pc2113 (Figure 3A). All *Phytophthora* spp. compared shared a total of 7,457 orthogroups, which represent ~51% of the total proteins in Pc2113 (*n* = 11,030) and Pc2109 (*n* = 10,904) (Figure 3B). In addition, 2,540 of these orthogroups were single-copy “core” orthologs. All the proteins and their corresponding encoding genes shared among all genomes analyzed will be referred to as “OG7457” for the remainder of this article. A total of 322 orthogroups are shared among all the *Phytophthora* spp. analyzed, except for the California *P. cinnamomi* isolates. Interestingly, Pc2109 and Pc2113 contain 904 orthogroups that are not present in the other *Phytophthora* spp. analyzed, representing ~6.2% of their proteomes (1,349 and 1,308 for Pc2113 and Pc2109, respectively). Proteins that belong to these orthogroups will be referred to as “OG904” for the remainder of this article. A total of 148 and 136 orthogroups were also identified only in Pc2113 (386 proteins) and in Pc2109 (362 proteins), respectively. To determine the total number of proteins only present in each isolate, we combined these proteins with the Pc2113 and Pc2109 unassigned proteins. After removing positive hits using tBLASTn and Exonerate, we calculated a total of 1,019 and 1,165 proteins present only in Pc2109 (OG-Pc2109) and Pc2113 (OG-Pc2113), respectively.

### 3.4 California *P. cinnamomi* isolates conform to the two-speed genome model with a larger effector repertoire in the Pc2109 fast-evolving plastic genome region when compared with Pc2113

The optimal L value for delimiting the genome into gene-dense and gene-sparse regions was determined to be 1.1 Kb (Supplementary Figures S4A, B). Delimitation of the genomes with an FIR L value of 1.1 Kb yielded 8,281 (38.7%) and 7,849 (37.2%) genes in GDRs for Pc2113 and Pc2109, respectively. Approximately 37% of the genes from Pc2113 (8,030) and Pc2109 (7,853) resided in the in-between regions, and 18% of Pc2113 (3,938) and Pc2109 (3,970) genes were in GSRs. The positions of < 7% of Pc2113 (1,153) and Pc2109 (1,439) genes could not be determined (ND) (Supplementary Figures S4C, D). The genome delimitation allowed us to determine the distribution of predicted genes associated with pathogenicity and virulence within these regions. Functional annotation revealed that Pc2109 encoded slightly more NIPs and elicitins than Pc2113, but fewer CAs and CRNs. Overall, regions where these effectors resided were consistent among the two isolates; however, Pc2109 displayed slightly higher effector counts within GSRs than Pc2113, except for the elicitins. A higher proportion of predicted NIPs (~50%), CAs (~50%), elicitins (~46%), and CRN (~34%) shared orthology with other *Phytophthora* spp. (OG7457) (Table 2). A total of 632 and 583 effectors were predicted in Pc2109 and Pc2113 genomes, respectively, using EffectorP. The predicted subcellular localization of these effectors was similar for both isolates; however, Pc2109 exhibited more apoplastic elicitins. After excluding annotated/predicted elicitins, NLPs, CWDEs, CAs, RXLRs, and CRNs from the EffectorP predictions, a total of 332 and 333 effectors were identified for Pc2109 and Pc2113, respectively (Supplementary Table S3, Table 2).

**Table 2:** Count table of predicted gene families encoding different proteins involved in pathogenicity in Pc2113 and Pc2109.

**Isolate**	**Gene family**	**Total proteins†**	**Orthologs shared with *Phytophthora* spp**.	**Region**	**EffectorP^♢^**
				**GDR | GSR | In-between | ND**	**A | C | D**
Pc2113	NIP	27	15	2 | 13 | 11 | 1	0 | 2 | 0
	Elicitins	43	23	15 | 7 | 19 | 2	11 | 2 | 0
	Carbonic Anhydrases	28	14	4 | 8 | 14 | 2	1 | 4 | 0
	Crinklers	28	6	1 | 8 | 16 | 3	0 | 1 | 0
Pc2109	NIP	34	17	3 | 17 | 8 | 6	0 | 1 | 0
	Elicitins	47	22	16 | 5 | 25 | 1	22 | 2 | 0
	Carbonic Anhydrases	26	16	4 | 9 | 11 | 2	1 | 5 | 0
	Crinklers	29	10	1 | 13 | 14 | 1	0 | 2 | 0

Region denotes whether the gene resides in GDRs, GSRs, in-between, or not-determined (ND) regions. ^†^ Counts include alternative transcripts. ^♢^Effector P 3.0 prediction; A, Apoplastic; C, Cytoplasmic; D, Dual-localized.

### 3.5 California *P. cinnamomi* isolates encode large repertoires of CAZymes and RXLR effectors

CAZyme prediction yielded 378 and 372 putative CAZymes for Pc2113 and Pc2109, respectively (Supplementary Table S4). Isolate Pc2113 encoded more CWDEs (299) compared to Pc2109 (276). For both isolates, the GH family made up the majority of the predicted CWDEs, followed by the PL family. In both isolates, ~60% of CWDEs were common to other *Phytophthora* spp. (OG7457), and ~30% resided in GSRs. We identified several CWDEs that were present in the California *P. cinnamomi* isolates (OG904), with more than 50% residing in the GSRs. Isolate Pc2109 contained a higher proportion of private CWDEs (OG-Pc2109) in GSRs than Pc2113 (OG-Pc2113).

**Figure 4:**
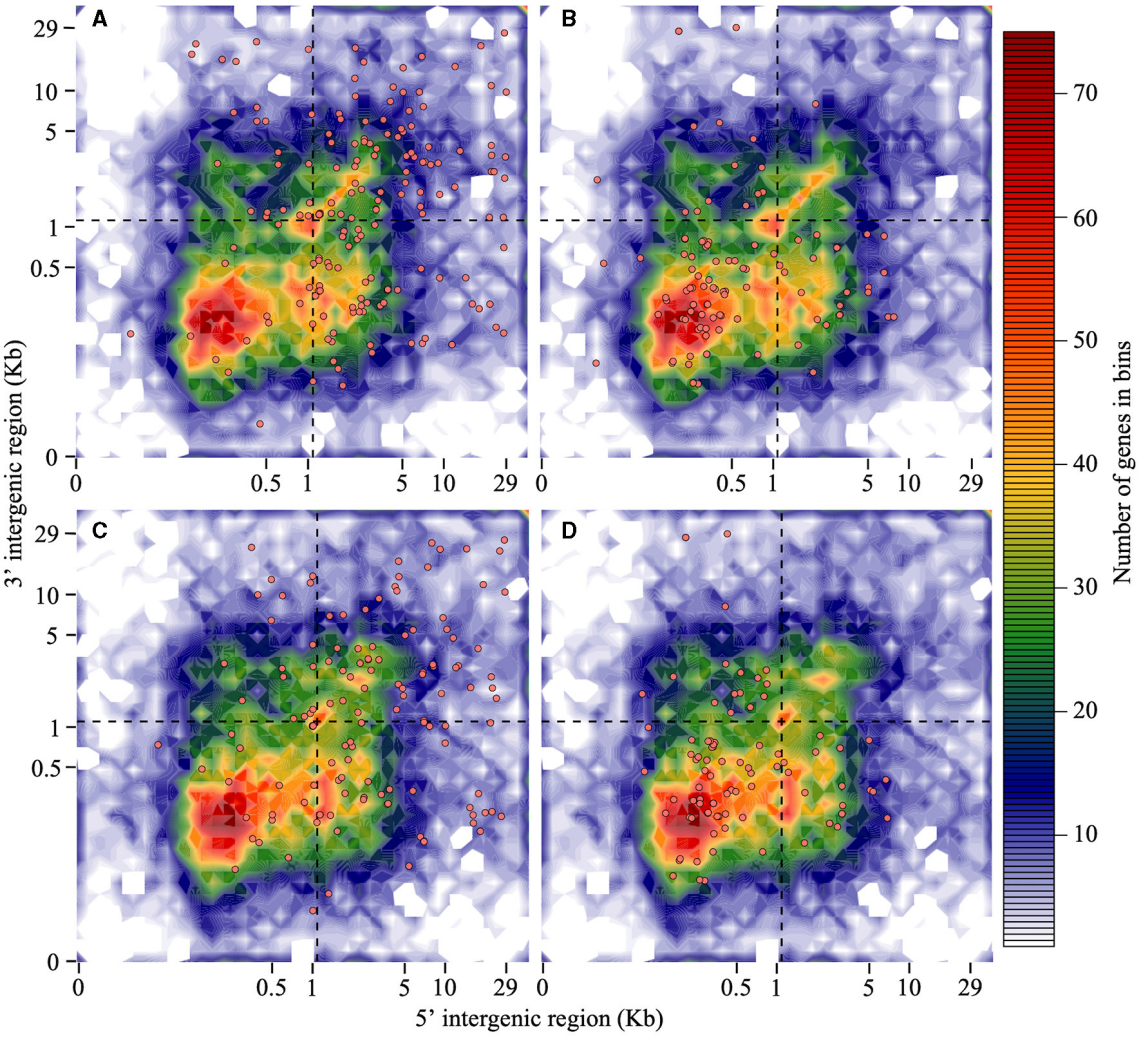
RXLRs are mainly located in the gene-sparse region of the *P. cinnamomi genome* when compared with housekeeping genes. Distribution of Pc2113 and Pc2109 RXLR effector genes and housekeeping ribosomal protein genes (the 60S and 40S) according to the length of their 5′-FIR (*x*-axis) and 3′-FIR (*y*-axis) is shown. The dashed line at 1.1 Kb (optimal L value) delimits the genome into GDR, GSR, and in-between regions. Distribution patterns of Pc2109 **(A)** and Pc2113 **(C)** RXLR effectors and Pc2109 **(B)** and Pc2113 **(D)** housekeeping ribosomal proteins are shown as orange-filled dots. Not all predicted RXLRs or housekeeping genes are displayed due to ND FIR boundaries.

Isolate Pc2109 harbors 222 predicted RXLRs, 49 more than Pc2113 (Supplementary Table S5). Approximately 22% of the predicted RXLR effectors in both isolates contain a putative WY-domain based on the HMMER search (E-value of < 0.05). For Pc2109, more effectors resided in GSRs (102) and in-between regions (84) when compared with Pc2113 encoding 74 and 64 in those regions, respectively. On the contrary, more Pc2113 effectors (23) resided in GDRs compared with Pc2109 (14). Less than 10% of the effector distributions for each isolate were ND (Figures 4A, C). As a control, the majority of the selected Pc2113 and Pc2109 housekeeping genes (the 40s and 60s ribosomal proteins) resided in GDRs, with ~1% in GSRs (Figures 4B, D). Approximately 22% and 6% of the RXLRs predicted from both isolates were also present in the OG7457 and the OG904 clusters, respectively (Supplementary Table S5). Orthology analysis of Pc2113, Pc2109, and GKB4 proteomes revealed several protein clusters specific to each isolate and 12,061 orthogroups shared among all isolates, representing < 10% and 76% of their proteomes, respectively (Supplementary Figure S3C). The clustering of the predicted CAZymes and RXLRs from the two California isolates and the GKB4 also revealed common and isolate-specific orthogroups (Supplementary Figures S3D, E). We identified a cluster of 184 common orthogroups containing 362, 293, and 279 predicted CAZymes in GKB4, Pc2113, and Pc2109, respectively, representing ~75% of the total repertoire in each isolate. A total of 12 GKB4 orthogroups, corresponding to ~15% of the CAZyme repertoire, were not present in the California isolates. Only two Pc2113 orthogroups, corresponding to ~1% of the CAZyme repertoire, were not present in GKB4 or Pc2109 (Supplementary Figure S3D). Similarly, we found 45 orthogroups containing 89, 60, and 83 common RXLRs in GKB4, Pc2113, and Pc2109, respectively, representing ~34–49% of the total repertoire in each isolate. A total of nine, seven, and two orthogroups were only found in GKB4, Pc2109, and Pc2113 isolates, respectively. GKB4 and Pc2109 exhibited more private RXLRs (13%) when compared with Pc2113 (8%) (Supplementary Figure S3E).

### 3.6 *Phytophthora cinnamomi* exhibited host-specific differential gene expression profiles during infection

Prior to RNA-seq, we performed detached leaf inoculations with Pc2113 infecting *A. thaliana, N. benthamiana*, and avocado and used trypan blue staining at 16, 24, and 90 hpi (*A. thaliana* only) to track Pc2113 infection (data not shown). The Pc2113 infection in *A. thaliana* at 90 hpi was consistent with the Pc2113 infection at 24 hpi in *N. benthamiana* and avocado, which led us to include the 90 hpi time point for *A. thaliana*. Following, we conducted an RNA-seq experiment to evaluate the expression patterns of Pc2113 when infecting *A. thaliana, N. benthamiana*, and avocado at 16, 24, and 90 hpi. Pathogen reads mapped ~91% and < 8% in Pc2113 growing *in vitro* and *in planta*, respectively. Interestingly, Pc2113 inoculation produced different lesions in each host evaluated, with more chlorotic and less necrotic lesions observed in the most susceptible *A. thaliana* ecotype (Robinson and Cahill, 2003), followed by *N. benthamiana*, which displayed water-soaking-like necrotic lesions, and avocado, which exhibited darker necrotic lesions. Lesion progression in each host was correlated with the increase of Pc2113 biomass in the infected tissue. In addition, the mapping percentage in each host increased over time and was correlated with the lesion phenotypes in each host (Supplementary Figure S5, Supplementary Table S6).

**Figure 5:**
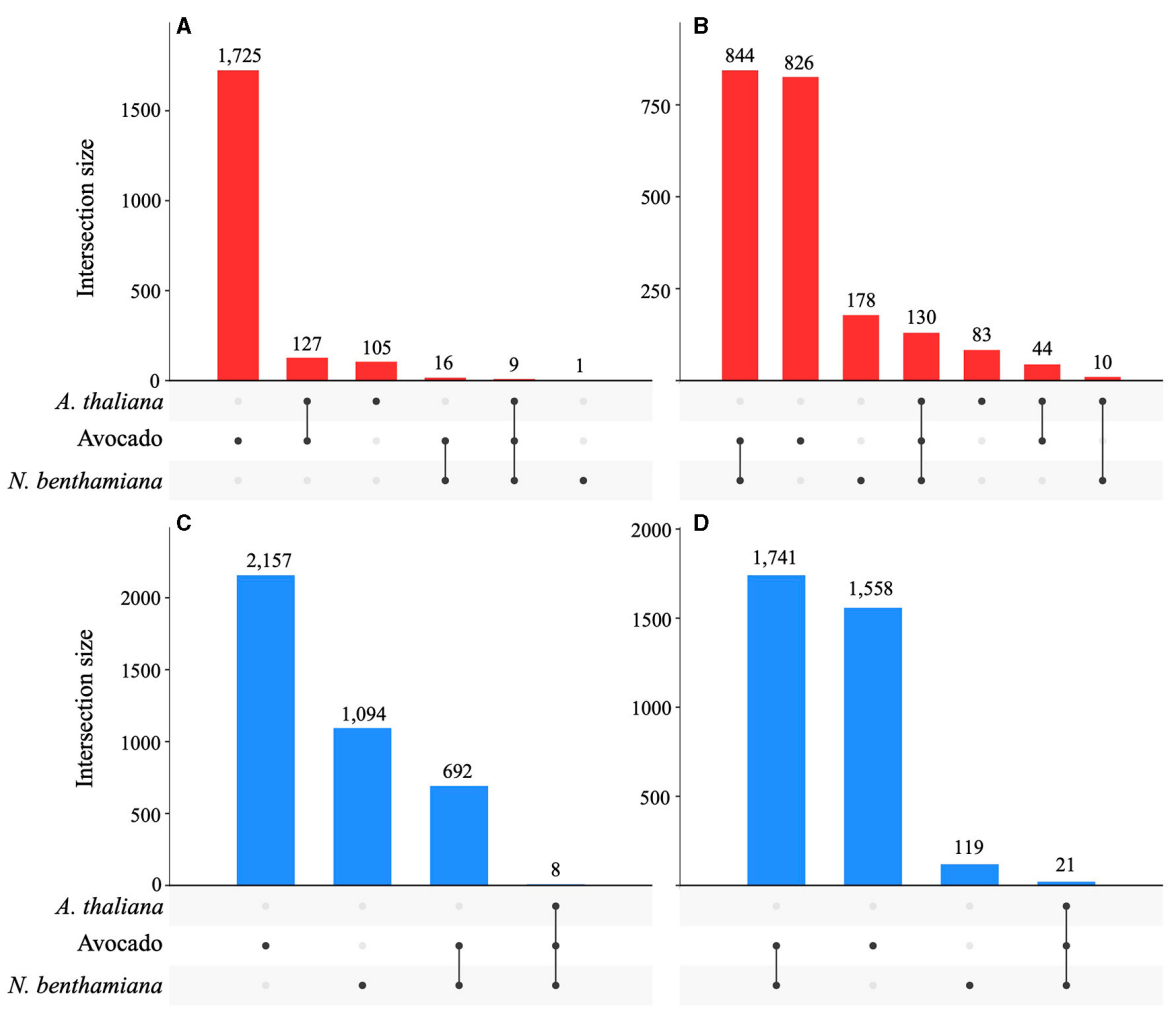
*Phytophthora cinnamomi* deploys common and different subsets of genes depending on the host infected. Upset plots indicating the number of common and host-specific differentially expressed genes (|LFC| ≥ 2, *p* < 0.05) in Pc2113 infecting *A. thaliana*, avocado, and *N. benthamiana* at 16 hpi and 24 hpi. The numbers of upregulated genes at 16 hpi **(A)** and 24 hpi **(B)** and downregulated genes at 16 hpi **(C)** and 24 hpi **(D)** are indicated at the top of each bar.

A total of 872 DEGs were identified across all timepoints in *A. thaliana* (546 up- and 326 down-regulated), 4,224 DEGs in *N. benthamiana* (1,162 up- and 3,048 downregulated), and 5,693 DEGs in avocado (2,048 up- and 3,645 downregulated). In avocado, 58% of the Pc2113 DEGs shared orthology with other *Phytophthora* spp. (OG7457), compared with 66% of the Pc2113 DEGs in *A. thaliana* and *N. benthamiana*. Less than 5% of the Pc2113 DEGs in each host belonged to the OG904 or OG-Pc2113 clusters. The distribution of Pc2113 DEGs that resided in GSRs was similar in all hosts analyzed (~17%) (Supplementary Table S7). A total of 249, 1,820, and 5,113 Pc2113 DEGs were identified in *A. thaliana, N. benthamiana*, and avocado, respectively, at 16 hpi, increasing over time, reflecting the disease development and lesion appearance when Pc2113 infected each host (Supplementary Figure S5). We observed a slight decrease in the number of Pc2113-upregulated genes in avocado and *A. thaliana* at the latest time point analyzed (Supplementary Table S8). RNA-seq analyses revealed Pc2113 DEGs in all hosts, two hosts, and specific hosts at 16 and 24 hpi (Figure 5). We found nine and eight Pc2113 genes significantly up- and down-regulated at 16 hpi (Figures 5A, C) and increased to 130 up- and 21 down-regulated at 24 hpi in all hosts, respectively (Figures 5B, D). There were more common subsets of upregulated genes between the two hosts, while only *N. benthamiana* and avocado shared overlapping sets of downregulated genes. Several subsets of upregulated genes decreased over time in *A. thaliana* and avocado but increased in *N. benthamiana* (Figures 5A, B). Similarly, the amount of downregulated genes decreased over time in *N. benthamiana* and avocado; however, no downregulated genes were found only in *A. thaliana* (Figures 5C, D).

### 3.7 *Phytophthora cinnamomi* deploys various subsets of differentially expressed necrosis-inducing proteins (NIPs), elicitins, and carbonic anhydrases (CAs) when infecting different hosts

Necrosis-inducing proteins, elicitins, and CAs have been shown to play important pathogenicity roles in several *Phytophthora*–plant interactions (Raffaele et al., 2010; Reitmann et al., 2017; Hardham and Blackman, 2018). The differential expression of these effectors varied among the hosts analyzed, with the least exhibited in *A. thaliana*, followed by *N. benthamiana* and avocado. Approximately 12, 8, and 1 of the predicted NIPs were upregulated when infecting avocado, *N. benthamiana*, and *A. thaliana*, respectively. The NIP upregulated in *A. thaliana* (Pc2113T1C00002230g0000120.1) was also upregulated in the other hosts. Interestingly, four of the NIPs were specifically upregulated in avocados. Only two NIPs were downregulated in avocado and no other hosts. Similarly, 17, 9, and 6 of the elicitins were upregulated and 7, 6, and 2 were downregulated in avocado, *N. benthamiana*, and *A. thaliana*, respectively. Four elicitins were found to be exclusively upregulated *in planta* (Pc2113T1C00002373g0000080.1, Pc2113T1C00002392g0000050.1, Pc2113T1C00002418g0002680.1, and Pc2113T1C00002607g0000450.1). Several elicitins exhibited host-specific DE in avocado, including the upregulated Pc2113T1C00002583g0000030.1 (OG904) and the downregulated Pc2113T1C00002220g0000590.1. The Pc2113T1C00002220g0000580.1 elicitin was only downregulated in *N. benthamiana*. Different subsets of CAs were DE, with 7, 4, and 2 upregulated and 5, 5, and 1 downregulated in avocado, *N. benthamiana*, and *A. thaliana*, respectively. The CA, Pc2113T1C00003123g0000220.1, was upregulated exclusively in avocados. Furthermore, none of the DE NIPs, elicitins, or CAs were *A. thaliana*-specific. The number of up- and down-regulated genes reported as host-specific is limited to those DE *in planta* (not DE in the zoospore stage) (Supplementary Table S7).

### 3.8 Cell wall-degrading enzymes exhibit host-specific differential expression patterns

**Figure 6:**
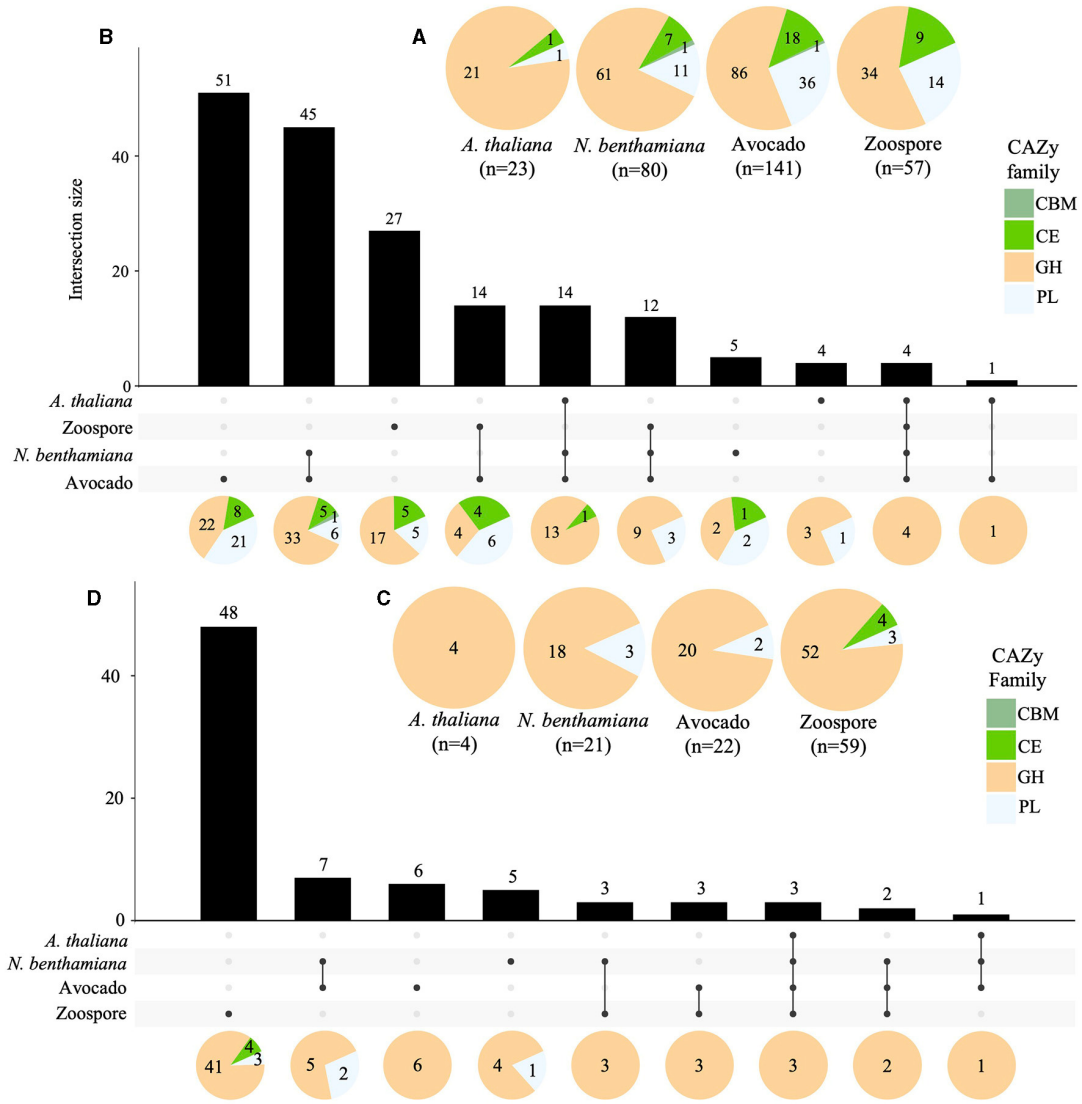
Regulation of Pc2113 CWDEs in all hosts across all timepoints tested. Pie charts displaying the total number of upregulated **(A)** and downregulated **(C)** Pc2113 CWDEs and their respective distribution by CAZy family for each host. Overlapping and host-specific upregulated **(B)** and downregulated **(D)** CWDEs are displayed as upset plots. Distribution of CWDEs by CAZy family are also displayed at the bottom as pie charts.

A total of 141, 80, and 23 of the predicted CWDEs (299) were upregulated in avocado, *N. benthamiana*, and *A. thaliana*, respectively. Additionally, 57 CWDEs were upregulated in zoospores. The majority of the upregulated CWDEs belonged to the GH CAZy family, followed by the PL family, except for *A. thaliana*, with only one PL upregulated. Only one member of the CBM CAZy family was upregulated in avocado and *N. benthamiana* (Supplementary Table S7, Figure 6A). A total of 14 CWDEs were upregulated in all hosts (13 GH and one CE). Several CWDEs were upregulated between two hosts, except for *A. thaliana* and *N. benthamiana*. A total of 45 CWDE were commonly upregulated in avocado and *N. benthamiana* (33 GH, 5 CE, 6 PL, and 1 CBM) and one GH in avocado and *A. thaliana*. A total of 51 CWDEs were exclusively upregulated in avocado, with 42 upregulated at both timepoints and 17 located in GSRs. Several CWDEs belonged to the OG7457 clusters (23) and two PLs, Pc2113T1C00000567g0000150.1 and Pc2113T1C00001098g0000110.1, to the OG904 and OG-Pc2113 clusters, respectively. Five CWDEs were exclusively upregulated at 24 hpi when infecting *N. benthamiana*, with all but one CE (Pc2113T1C00002636g0002400.1) located in GSRs. Only two, Pc2113T1C00002589g0001670.1 (PL) and Pc2113T1C00002397g0000460.1 (GH), shared orthology with *Phytophthora* spp. (OG7457). We identified four CWDEs exclusively upregulated in *A. thaliana*, including three GHs and one PL. Only two *A. thaliana*-specific CWDEs (GHs) resided in GSRs and shared orthology with *Phytophthora* spp. (OG7457) (Supplementary Table S7, Figure 6B).

In contrast to the upregulated CWDEs, there were fewer downregulated CWDEs in avocado (22), *N. benthamiana* (21), and *A. thaliana* (4), with the majority in the GH CAZy family. No downregulated CWDEs in the CBM family were detected, and members of the CE family were only found downregulated in zoospores (Supplementary Table S7, Figure 6C). A GH CWDE (Pc2113T1C00002345g0000380.1) was downregulated in all hosts. Only avocado and *N. benthamiana* shared seven downregulated CWDEs. Of these seven, two of the GHs shared orthology with *Phytophthora* spp. (OG7457), one PL (Pc2113T1C00001147g0000260.1) belonged to the OG904 cluster, and another PL (Pc2113T1C00002233g0000020.1) to the OG-Pc2113 cluster. Five CWDEs were exclusively downregulated in *N. benthamiana*. Of these five, only two of the GHs shared orthology with *Phytophthora* spp. (OG7457). Interestingly, the GH (Pc2113T1C00002958g0000060.1) and PL (Pc2113T1C00002426g0000100.1) enzymes were found to be exclusively upregulated in avocado and *A. thaliana*, respectively, but downregulated in *N. benthamiana*. Six CWDEs were exclusively downregulated in avocado, and four belonged to the OG7457 cluster. We did not detect any downregulated CWDEs exclusive to *A. thaliana* (Supplementary Table S7, Figure 6D).

### 3.9 Differential expression and phylogenetic analysis of *P. cinnamomi* RXLRs reveal evolutionary relatedness and various host-specific expression patterns

Differential RXLR expression was detected when infecting different hosts. Approximately 27% (47) and 6.9% (12) of the predicted Pc2113 RXLRs (173) were up- and down-regulated across all samples and timepoints, respectively. In *A. thaliana*, the number of upregulated RXLRs decreased over the time course of the infection. Three out of the seven upregulated RXLRs in this host belonged to the OG7457 (Pc2113RXLR061, Pc2113RXLR098, and Pc2113RXLR092) and one (Pc2113RXLR047) to the OG904 clusters. No downregulated RXLRs were detected in this interaction. When infecting *N. benthamiana*, 10.4% (18) and 3.4% (6) RXLRs were up- and down-regulated during the infection, respectively. Of these upregulated RXLRs, seven resided in GSRs, seven shared orthology with *Phytophthora* spp. (OG7457), and two (Pc2113RXLR047 and Pc2113RXLR052) belonged to the OG904 clusters. There were two downregulated RXLRs in GSRs; one belonged to the OG7457 clusters, and two (Pc2113RXLR149 and Pc2113RXLR150) to the OG904 clusters. A total of 37 and 12 RXLRs were up- and down-regulated in avocados, respectively. In total, 15 of these upregulated RXLRs resided in GSRs; 13 belonged to the OG7457 clusters, and two (Pc2113RXLR047 and Pc2113RXLR052) to the OG904 clusters. Four downregulated RXLRs in avocado resided in GSRs, four shared orthology with *Phytophthora* spp. (OG7457), and two (Pc2113RXLR149 and Pc2113RXLR150) belonged to the OG904 clusters. We found 39 DE RXLRs (16 up- and 23 down-regulated) in zoospores. A total of 4 up- and 10 down-regulated RXLRs shared orthology with *Phytophthora* spp. (OG7457). In addition, three up- and two down-regulated RXLRs belonged to the OG904 cluster, and one downregulated RXLR (Pc2113RXLR077) belonged to the OG-Pc2113 clusters. A total of 31 and 3 RXLRs were exclusively up- and down-regulated *in planta*. There were RXLR subsets that were DE in all hosts, and some were host-specific. In total, 3 RXLRs were upregulated in all hosts (Pc2113RXLR047, Pc2113RXLR096, and Pc2113RXLR092), 11 were common to *N. benthamiana* and avocado, and 1 RXLR was overlapping between *A. thaliana* and avocado. We found one RXLR upregulated only in *N. benthamiana* and 15 in avocado. Interestingly, none of the downregulated RXLRs were commonly expressed in all hosts or between two hosts, and only three RXLRs were significantly downregulated in avocados (Supplementary Table S7).

**Figure 7:**
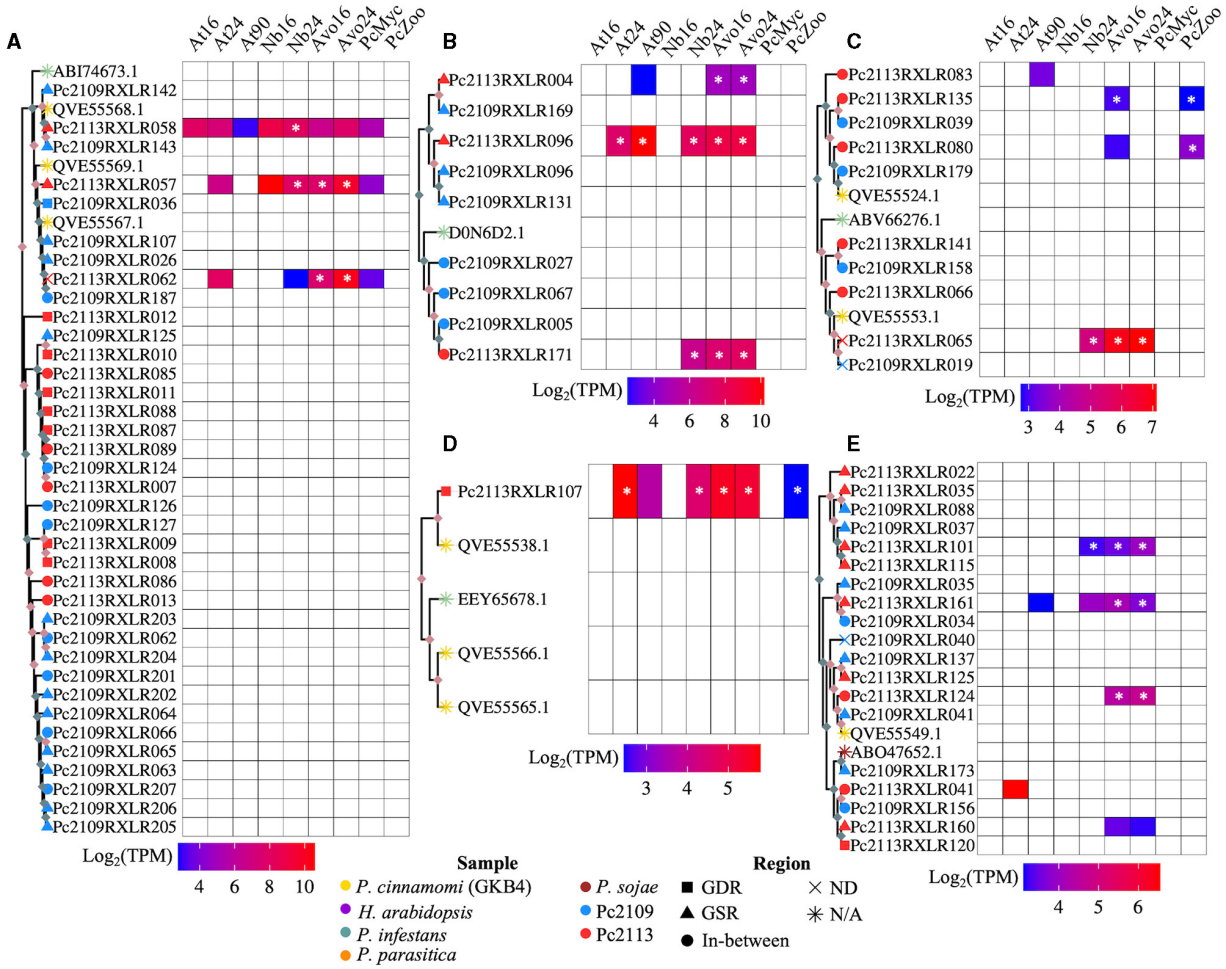
Subclades of the RXLR phylogenetic tree and a heatmap of the expressed RXLRs log_2_(TPM). **(A)** A subclade of RXLRs where the top portion of the tree contains SNE1 RXLRs from P. cinnamomi isolates Pc2113, Pc2109, and GKB4 as well as *P. infestans*. The bottom portion is exclusively made up of Pc2113 and Pc2109 RXLRs in which both isolates exhibit tandem-duplicated RXLRs with Pc2109 encoding more and with a higher proportion residing in GSRs than Pc2113. **(B)** A subclade made up of mostly Pc2113 and Pc2109 RXLRs and one *P. infestans* RXLR (Haas et al., 2009; Oh et al., 2009; He et al., 2019). All the Pc2113 RXLRs are exclusively upregulated *in planta* with two residing in GSRs and Pc2109 demonstrates an expansion of these phylogenetically related RXLRs compared to Pc2113. **(C)** A subclade consisting of RLXRs from *P. cinnamomi* isolates Pc2113, Pc2109 and GKB4, and one *P. infestans* RXLR encoded as avr4 (van Poppel et al., 2008). Several of the Pc2113 RXLRs within this clade are differentially expressed exclusively *in planta*, in the zoospore stage, or both. **(D)** Subclade of *P. cinnamomi* RXLRs and *P. infestans* RXLR. The Pc2113 RXLR resides in GDRs and is differentially expressed both *in planta* and in the zoospore stage. **(E)** Subclade of RXLRs made up of *P. cinnamomi* RXLRs and one *P. sojae* RXLR. Majority of the California *P. cinnamomi* RXLRs reside in GSRs and many of the differentially expressed Pc2113 RXLRs are exclusive to avocado. *Indicates significantly differentially expressed compared to somatic mycelium. Nodes without ultrafast bootstrap values inherit the value from the preceding node because they are identical sequences. Ultrafast bootstrap values over 95 are denoted by light pink diamonds and values under 95 are denoted by light blue diamonds. The sample is denoted by the branch tip color and the shape denotes which region of the genome the RXLR resides, if applicable.

Phylogenetic analysis revealed the evolutionary relatedness of the Pc2113 and Pc2109 RXLRs, along with previously characterized RXLRs from various *Phytophthora* spp. (Figure 7, Supplementary Figure S6). Several *P. cinnamomi* RXLRs effectors from this study clustered with a *P. infestans* suppressor of necrosis 1 (SNE1) effector (NCBI accession number ABI74673.1) (Kelley et al., 2010). This cluster contained three Pc2113 RXLRs (Pc2113RXLR058, Pc2113RXLR057, and Pc2113RXLR062) annotated as SNE1, with two that resided in the GSR, and all Pc2109 RXLRs in this cluster resided in GSRs and were annotated as SNE1, except for Pc2109RXLR187. Pc2113RXLR058 was upregulated only in *N. benthamiana*, Pc2113RXLR057 was upregulated in avocado and *N. benthamiana*, and Pc2113RXLR062 was exclusively upregulated in avocado. This SNE1 clade is closely related to another clade containing several Pc2113 and Pc2109 RXLRs annotated as “unknown proteins”. In this clade, we observed an expansion of RXLRs in both Pc2113 and Pc2109. For Pc2109RXLR125, we observed an expansion of RXLRs in Pc2113 (7). Similarly, we observed a larger cluster of Pc2109 RXLRs (12) with the majority in GSRs (8) and the remaining four in the in-between regions (Figure 7A). Another RXLR clade grouped several Pc2113 and Pc2109 RXLRs with a characterized *P. infestans* suppressor of plant immunity RXLR (NCBI accession number D0N6D2.1) (Zheng et al., 2014; He et al., 2019). The subclade contained D0N6D2.1 and was composed of one putative homolog in Pc2113 and three homologs in Pc2109. Pc2113RXLR171 from this cluster was upregulated in avocado and *N. benthamiana*. Associated with D0N6D2.1, we identified another subclade composed of two RXLRs (RXLR004 and RXLR169) annotated as “Avirulence protein 1b” and three RXLRs (RXLR096s and RXLR131) annotated as “Avr1b Avirulence-like protein”, all located in GSRs in both genomes. Pc2113RXLR004 was exclusively upregulated in avocados, and Pc2113RXLR096 was upregulated in all hosts (Figure 7B). We found a subclade that contained RXLRs related to previously annotated *P. cinnamomi* (NCBI accession numbers QVE55524.1 and QVE55553.1) (Joubert et al., 2021) and *P. infestans* Avr4 (NCBI accession number ABV66276.1) (van Poppel et al., 2008). This clade was further divided into two subclades: one subclade on top contained three Pc2113 RXLRs and two Pc2109 RXLRs related to QVE55524.1. Two Pc2113 RXLRs (Pc2113RXLR135 and Pc2113RXLR080) were upregulated in zoospores, and Pc2113RXLR135 was also upregulated in avocado at 16 hpi. The bottom subclade was made up of *P. cinnamomi* RXLRs annotated as ‘Avr4′ with one member, Pc2113RXLR065, upregulated only in avocado and *N. benthamiana* (Figure 7C). We identified clades that contained previously annotated RXLRs from *P. cinnamomi* (NCBI accession numbers QVE55566.1, QVE55565.1, and QVE55538.1) (Joubert et al., 2021) and *P. infestans* (NCBI accession number EEY65678.1) (McLellan et al., 2013). Interestingly, no homologs or few homologs to those RXLRs were found in Pc2113 or Pc2109. One QVE55538.1 homolog was identified in Pc2113 (Pc2113RXLR107) and was upregulated in all samples (Figure 7D). A clade that contained several Pc2113 and Pc2109 RXLRs related to *P. sojae* Avr1a (NCBI accession number ABO47652.1) was identified; however, none of these RXLRs were annotated as Avr1a homologs. Pc2113RXLR124 and Pc2109RXLR041 were annotated as “Avr1b-1 avirulence-like protein” and were grouped with a previously annotated *P. cinnamomi* RXLR (NCBI accession number QVE55549.1). Pc2113RXLR124 was found to be exclusively upregulated in avocados. Pc2113RXLR101 and Pc2113RXLR115 were annotated as “avh87”, and only Pc2113RXLR101 exhibited upregulation in avocado and *N. benthamiana*. Pc2109RXLR35, Pc2109RXLR34, and Pc2113RXLR161 were annotated as “Avh242”, and only Pc2113RXLR161 exhibited upregulation in avocado (Figure 7E).

### 3.10 Global expression of effectors confirms *Phytophthora cinnamomi's* strategies when infecting different hosts

To confirm the host-specific patterns of the *P. cinnamomi* effectors exhibiting significant up- or down-regulation in this study, we assessed the overall gene expression profiles in terms of TPM of 18 CAs, 220 CWDEs, 34 elicitins, 22 NIPs, and 81 RXLRs in all samples. Consistent with the DE analyses, global expression indicated that *P. cinnamomi* expressed (TPM > 5) fewer effectors when infecting *A. thaliana* and *N. benthamiana* at 16 hpi when compared with *N. benthamiana* at 24 hpi and avocado. Interestingly, more effectors were expressed *in planta* and located in the GSRs when compared with their expression in *P. cinnamomi* growing *in vitro*, especially for CWDEs, RXLRs, and elicitins (Supplementary Figure S7 and Supplementary Table S9). These results are consistent with the lesion phenotypes of the pathogen infecting each host (Supplementary Figure S5). We selected three effectors and validated their expression in *N. benthamiana* leaves infected with *P. cinnamomi* using qPCR analyses. Consistent with our RNA-seq expression data, Pc2113RXLR047 and Pc2113RXLR52 were highly upregulated at 16 and 24 hpi when compared with the mycelia and sporangia samples from the pathogen growing *in vitro*. Similar results were found for the elicitin (Pc2113T1C00002373g0000080.1) expression assessed by qPCR, showing significant upregulation at 16 and 24 hpi (Supplementary Figure S8).

## 4 Discussion

We generated two additional high-quality genome assemblies of two genetically and phenotypically distinct *P. cinnamomi* isolates associated with Avocado PRR in California. Our comparative genomic study, coupled with the transcriptome analyses of *P. cinnamomi* during infection of several hosts, uncovered the existence of common and host-specific expression patterns, providing insights into the different *P. cinnamomi* infection strategies. Here, we reported the largest *P. cinnamomi* genome assemblies to date and to the best of our knowledge, the first guided by flow cytometry (Studholme et al., 2016; Longmuir et al., 2017; Engelbrecht et al., 2021). Despite our efforts to remove heterozygous contigs, BUSCO analysis still displayed a larger proportion of duplications in both genomes when compared with all publicly available *P. cinnamomi* genomes (Table 1) (Engelbrecht et al., 2021). To date, the recent GKB4 *P. cinnamomi* reference genome reported by Engelbrecht et al. (2021) and the assemblies reported in this study are the least fragmented genomes available, and they share high levels of macrosynteny and proteome orthology. We did not perform scaffolding; thus, our genomes are less contiguous than the GKB4 *P. cinnamomi* isolate; however, our BUSCO values, number of predicted genes, and secreted proteins were similar (Engelbrecht et al., 2021). Interestingly, the more virulent isolate, Pc2109, encoded the largest reported *P. cinnamomi* secretome. Furthermore, this Pc2109 isolate exhibited an expanded repertoire of RXLRs when compared with the less virulent isolate, Pc2113, consistent with the increased number of tandem insertions found in this isolate. The expansion of genes associated with pathogenicity, such as RXLRs and CWDEs, could be one explanation for the increased duplications in our assemblies reflecting the adaptation of this pathogen to the several commercially available resistant rootstocks used in California. Moreover, previous studies characterizing A2 *P. cinnamomi* isolates associated with avocado PRR revealed low levels of genetic differentiation and large phenotypic variation among clonal isolates (Pagliaccia et al., 2013; Belisle et al., 2019a,b). A troubling feature of invasive alien species, such as *P. cinnamomi*, is the ability to successfully establish, adapt, and proliferate in new environments due to clonality; thus, duplication events, gene expansion, and polyploidization or aneuploidy could explain this large phenotypic variability in *P. cinnamomi* clonal populations in California.

Polyploidization has been suggested as a mechanism for increasing fitness and adaptation in asexual populations of *Phytophthora* spp. (Li et al., 2017; Shrestha et al., 2017; Dale et al., 2019), including *P. cinnamomi* (Calle-Henao et al., 2020; Engelbrecht et al., 2021, 2022). Several publications have reported that *P. cinnamomi* field isolates associated with avocado PRR in Colombia and South Africa exhibited various levels of ploidy and aneuploidy (Calle-Henao et al., 2020; Engelbrecht et al., 2021, 2022). Consistent with these reports, the ploidy estimation for the *P. cinnamomi* isolates in this study was triploid (Supplementary Figures S1, S2); however, we do not dismiss the possibility of aneuploidy as reported by Engelbrecht et al. (2021).

Across the diverse *Phytophthora* spp. proteomes analyzed, we found several orthogroups with the highest number of shared proteins (OG7457), suggesting that these may be “core” orthologs. Interestingly, a larger proportion of DE effectors after pathogen infection were found in these OG7457 orthogroups, implying that some of these effectors may play a conserved role in *Phytophthora* pathogenicity. Conserved orthogroups were only present in both Pc2113 and Pc2109 isolates (OG904) and specific orthogroups per isolate (OG-Pc2113 and OG-Pc2109) were also detected in this study. These findings align with previous studies that identified “core” orthologs shared among various oomycetes, species-specific, and isolate-specific orthologs (Fletcher et al., 2018, 2023; Dussert et al., 2019; Ayala-Usma et al., 2021; Gogoi et al., 2023). Moreover, orthology analyses among the California *P. cinnamomi* isolates and GKB4 also revealed ‘core' and isolate-specific orthogroups (Supplementary Figure S3). The identification of Pc2113-, Pc2109-, and GKB4-specific and core orthogroups may provide insights into the phenotypic variability, including virulence, found among these isolates (Belisle et al., 2019a,b), as was seen in *P. cactorum* (Gogoi et al., 2023). To better define *P. cinnamomi*-specific orthogroups, a comprehensive orthology analysis using the available proteomes of each *Phytophthora* spp. needs to be conducted.

Many fungal and oomycete pathogens exhibit a genome architecture that conforms to the “two-speed genome” model, characterized by a bipartite structure with a core genome (GDR) and gene-sparse/repeat-rich regions (GSR) that serve as a cradle for adaptive evolution (Haas et al., 2009; Dong et al., 2015). Here, we confirmed the Pc2113 and Pc2109 two-speed genome architecture by delimiting their genomes into GDR, GSR, and in-between regions using single-copy ‘core” orthologs (Supplementary Figure S4) (Raffaele et al., 2010; Rojas-Estevez et al., 2020). To the best of our knowledge, these are the first *P. cinnamomi* genomes that have been successfully delimited into these regions. Interestingly, the proportions of proteins residing in these genome regions were like those reported for other *Phytophthora* spp., including *P. infestans* (Raffaele et al., 2010)*, P. betacei* (Rojas-Estevez et al., 2020), and *P. sojae* (Chen et al., 2018). In several plant and animal filamentous pathogens, this genome compartmentalization aids in the rapid evolution and diversification of pathogen effectors. Effector genes primarily localize within or adjacent to these rapidly evolving regions (GSRs), enabling pathogens to adapt to challenges such as host resistance, fungicides, and climate change, while essential genes are found in regions of relative genomic stability shielded from deleterious mutations (Sánchez-Vallet et al., 2018; Wacker et al., 2023). Consistent with this, we found many effectors from both isolates residing in GSRs with Pc2109 encoding more effectors within GSRs than Pc2113. The variation in the number and distribution of the Pc2109 effectors in GSRs could explain its high virulence phenotype. Consistent with this, Liu et al. (2016) found that more virulent *P. nicotianae* isolates encoded larger RXLR repertoires with a higher proportion in GSRs. The number of predicted RXLRs reported in the GKB4 isolate (Engelbrecht et al., 2021) is within the range of those found in our California isolates; however, fewer NIPs and CAZymes were predicted in these isolates when compared with GKB4. The NIPs in Pc2113 and Pc2109 align with the numbers reported by McGowan and Fitzpatrick (2017) for another isolate of *P. cinnamomi*, suggesting that the variation in the number of effectors may reflect the natural variation among *P. cinnamomi* isolates. Several studies have described “core” and “isolate-specific” effector repertoires in *Phytophthora* spp. (Liu et al., 2016; Zhang et al., 2019; Nellist et al., 2021). We compared the RXLR and CWDE repertoires of Pc2113, Pc2109, and GKB4 (Engelbrecht et al., 2021) and found that these effectors are part of both ‘core' and private orthogroups, with CWDEs exhibiting more “core” orthogroups than RXLRs (Supplementary Figures S3D, E).

Several RNA-seq studies have been performed for *P. cinnamomi* under various conditions, including avocado root infections (Engelbrecht et al., 2021), pre-infection structures (Reitmann et al., 2017), and stem infections of *Eucalyptus nitens* (Meyer et al., 2016); however, there are no reports assessing the infection of a single *P. cinnamomi* isolate infecting different hosts in the same RNA-seq study. Here, for the first time, we compared the gene expression profile of a single *P. cinnamomi* isolate (Pc2113) infecting *A. thaliana, N. benthamiana*, and avocado and provided valuable insights into common and host-specific infection strategies. We found subsets of Pc2113 DE genes in all hosts analyzed, suggesting a more conserved role during colonization and the existence of a core expression pattern independent of the host. Several subsets of Pc2113 DE genes also exhibited ‘host-specific' responses, suggesting a flexible specific pathogen response to different hosts. Our results are consistent with previous studies that also reported the existence of a “core” and host-specific expression patterns for other broad host range pathogens, including *Sclerotinia sclerotiorum* (Kusch et al., 2022)*, Fusarium graminearum* (Harris et al., 2016)*, P. cactorum* (Nellist et al., 2021), and *Myzus persicae* (Mathers et al., 2017), when colonizing different host plants. It was hypothesized that the existence of core expression patterns could target conserved plant genes, which could enable the pathogen for host jumps or host range expansions (Kusch et al., 2022). In addition, host-specific patterns will highlight the adaptation and pathogen strategies needed to colonize specific hosts. Consistent with this, we observed correlations between the number and expression patterns of Pc2113 DE genes, especially those encoding effectors with distinct lesion phenotypes (Supplementary Figures S5–S7).

Necrosis-inducing proteins have been reported to be expressed late during the colonization of soybeans by *P. sojae*, suggesting that NIPs induce necrosis to aid colonization during the necrotrophic stage of infection (Qutob et al., 2002). Consistent with this report, we noted a reduction in the number of NIPs upregulated in *A. thaliana*, which lacks the necrotic lesion phenotype, compared to *N. benthamiana* and avocado, where the number of upregulated NIPs increased and correlated with the lesion necrosis intensity. All the upregulated NIPs in *N. benthamiana* were also upregulated in avocados, suggesting a common role in triggering necrosis in these hosts. One NIP was found to be commonly upregulated in all hosts, suggesting a common function during *P. cinnamomi* colonization. The role of these NIPs in inducing necrosis and contributing to virulence needs to be further investigated. We detected subsets of “core” and host-specific upregulated Pc2113 elicitins. Elicitins are considered pathogen-associated molecular patterns (PAMPs) and are secreted in the apoplast of the infected cell, where they trigger defense responses including hypersensitive-like (HR) responses and systemic acquired resistance (Kamoun et al., 1993, 1998; Tyler, 2002; Derevnina et al., 2016). Recently, elicitins have been shown to enhance pathogenicity in certain plant–pathogen interactions (Kharel et al., 2021). Here, we reported several Pc2113 “core” elicitins exclusively upregulated *in planta* that share orthology with all *Phytophthora* spp. genomes analyzed (OG7457) and DE in all three hosts analyzed, suggesting an important role during plant–*P. cinnamomi* interactions. The roles of these elicitins during plant immunity or pathogenicity need to be further investigated.

Carbohydrate-active enzymes (CAZymes), especially CWDEs, have been shown to play critical roles during plant–pathogen interactions (Kubicek et al., 2014). Fungal and oomycete pathogens deploy a diverse range of CWDEs targeting each structural cell wall component to facilitate infection, colonization, and obtain nutrients (Kubicek et al., 2014; Blackman et al., 2015). We found that all the *P. cinnamomi* isolates compared in this study shared over 75% of their CAZymes repertoires. Moreover, over 50% of Pc2113 CWDEs differentially expressed after pathogen infection shared orthology with all *Phytophthora* spp. (OG7457) analyzed, highlighting the importance of these effectors during pathogen infection. The composition and structure of the cell wall differ significantly among plant lineages, growth, and development; thus, it is expected that wide host range pathogens, such as *P. cinnamomi*, encode a diverse arsenal of CWDE, which mirrors the complexity of the cell wall of the different hosts. Consistent with this hypothesis, we showed for the first time that the same *P. cinnamomi* isolate deployed a “core” and host-specific subsets of CWDEs. Moreover, the number of DE CWDEs correlated with the necrotic lesion phenotypes observed when infecting two herbaceous hosts (*A. thaliana* and *N. benthamiana*) and avocado, a woody-tree host with necrotic lesion phenotype exhibiting the higher number of DE CWDEs (Supplementary Figure S5 and Figures 6A, B). We found similar CWDE numbers, types, and 20 GKB4 CWDE homologs significantly upregulated in avocados to those reported by Engelbrecht et al. (2021), despite the differences in methods and infected avocado tissues, suggesting that CWDEs are crucial pathogenicity or virulence factors in *P. cinnamomi*. The majority of the CWDEs DE *in planta* found in this study belonged to the GH families, followed by PL and CE families. Glycoside hydrolases are abundantly secreted by oomycete and fungal pathogens, and their repertoire is linked to the pathogen lifestyle, with necrotrophic and biotrophic pathogens exhibiting the highest and smallest CWDE numbers and diversity, respectively (Kubicek et al., 2014; Saraiva et al., 2023). In agreement with these studies, we also found that Pc2113 deployed a larger number and diversity of GH families when infecting avocado and *N. benthamiana* than when infecting *A. thaliana* (chlorotic lesions).

Cellulose degradation involves the action of two types of cellulases (exocellulases and endocellulases), followed by β-glucosidases that hydrolyze cellodextrin oligomers to glucose. Exocellulases or cellobiohydrolases are GH6 and GH7 members. Interestingly, we did not find any exocellulases from these families of DE in *A. thaliana*; however, a higher number of GH6 and GH7 exocellulases were upregulated in avocado, followed by *N. benthamiana*. β-glucosidases are predominantly found in the GH1 and GH3 families. Interestingly, only one Pc2113 β-glucosidase (GH1) is DE (upregulated) in Arabidopsis. This GH1 is also upregulated in *N. benthamiana* and avocado, together with other GH1 and GH3 β-glucosidase members, suggesting that the GH1 upregulated in all hosts might play a more general role than pathogenicity. Several endo-β-1,4-glucanases and xyloglucan-specific endoglucanases belonging to the GH5 and GH12 families were commonly upregulated only *in planta* in all three hosts analyzed, suggesting a common role during *P. cinnamomi* pathogenicity. In addition, more GH5 and GH12 endocellulases were upregulated in *N. benthamiana* and avocado. Hemicellulose is an important part of the primary cell wall, and it has been shown that oomycete pathogens deploy xyloglucan-specific endoglucanases to promote host cell wall degradation. *Phytophthora sojae* XEG1 (GH12) was shown to be an important determinant of virulence in infected soybean roots (Ma et al., 2015; Xia et al., 2020). Recently, one *P. cinnamomi* xyloglucan-specific endo-β-1,4-glucanase 1 (GH12) was found to be upregulated in avocado roots infected with the GKB4 isolate (Backer et al., 2022).

After cellulose, xylan is the second most abundant complex and indigestible hemicellulose polysaccharide found in the cell wall. Plant pathogenic and saprophytic fungi produce a diverse number of CWDEs that degrade xylans, including xylanases (GH10, GH11, and GH30) and β-xylosidases (GH3, GH43, and GH54). In this study, we identified GH10, GH30, and GH43 xylan-degrading enzymes significantly upregulated in all hosts after Pc2113 infection; moreover, these CWDEs shared orthology with other *Phytophthora* spp. (OG7457). In addition, more GH30 members were commonly upregulated in avocado and *N. benthamiana*. However, arabinosidase GH43 and GH54 members were exclusively upregulated in avocados, suggesting that a diverse arsenal of CWDEs is needed to degrade a more complex cell wall found in woody hosts (e.g., avocado). Several studies have shown that knockdowns and disruptions of GH10 and GH11 members in fungal pathogens have significantly decreased fungal virulence (Nguyen et al., 2011; Van Vu et al., 2012). Two of the Pc2113 GH43 CWDEs upregulated in avocado in this study were homologs of the GKB4 GH43 CWDEs that were found to be upregulated in avocado-infected roots (Engelbrecht et al., 2021). Furthermore, one of these GH43 CWDEs also shared orthology with the *P. parasitica* PPTG_15711 enzyme, which was upregulated in infected lupine roots (Blackman et al., 2015), suggesting an important role of these GH43 CWDEs during *P. cinnamomi* pathogenicity that needs to be further investigated.

β-1,3 glucans are major components of callose depositions, a defense response produced at the site of infection by pathogens. Members of these GH72 and GH81 families have been found upregulated in *P. cinnamomi* GKB4 infecting avocado roots (Engelbrecht et al., 2021). Consistent with these findings, we found three Pc2113 GH72 and one GH81 CWDE member involved in β-1,3 glucan degradation commonly upregulated in all hosts, and some of them were upregulated at the zoospore stage. Our results support the hypothesis that these CDWEs could play dual roles during the *P. cinnamomi* infection process: one general role in *P. cinnamomi* cell wall development and modification and a second role during pathogenicity by degrading callose depositions produced as a defense response during cell wall penetration.

Degradation of cutin and pectin, plant cell wall constituents, needs the action of cutin hydrolases, polygalacturonases (PGs), and pectin/pectate lyases (PLs). Cutin hydrolases and pectinesterases have been assigned to the CE families, PGs to the GH28, and pectin/pectate lyases to the PL1, 3, and 9 families (Kubicek et al., 2014). GH28 PGs are the first enzymes to be secreted by fungal pathogens when they encounter the plant cell wall, and there are several reports arguing for their role as virulence factors in several necrotrophic pathogens (Shieh et al., 1997; Have et al., 1998; Isshiki et al., 2001; De Lorenzo and Ferrari, 2002; Oeser et al., 2002). Consistent with this, we found GH28 CWDEs only DE in *N. benthamiana* and avocado and not in *A. thaliana*; moreover, all these CWDEs also displayed upregulation in zoospores, indicating a basal level of these CWDEs in preparation for the plant infection as seen with fungal cutinases. Except for one Pc2113 pectinesterase (CE8), which was upregulated in all hosts, all CEs and PLs were DE in *N. benthamiana* and avocado. Moreover, Pc2113 exhibited a higher number of these upregulated CWDEs in avocados. Interestingly, the majority of the Pc2113 CWDEs belonging to the PL1 and CE families were exclusively DE in avocado, suggesting their roles during more necrotrophic *P. cinnamomic*–plant interactions. Consistent with these findings, a multitude of predicted CEs in fungal genomes were identified in *Macrophomina phaseolina*, one of the most destructive necrotrophic fungi infecting over 500 hosts (Su et al., 2001).

Overall, our results suggest that there is a *P. cinnamomi* “core” of CWDEs required for *P. cinnamomi* pathogenicity, while there are CWDE subsets of different families that could have been expanded to mediate host-specific infections. Consistent with this hypothesis, we identified several contigs containing genes in tandem duplications encoding for these CWDEs. For example, the contig Pc2113T1C00002491 contains five PL1s and four GH43 in tandem, with the exception of two, all of which were specifically upregulated in avocados (Supplementary Table S7). We found syntenic regions containing CWDEs (PL1_4s and GH43_6s) and Pc2113 homologs in tandem in the genomes of *P. parasitica* INRA-310 (scaffold NW_008649037.1) and *P. cinnamomi* GKB4 (scaffold JAFJYM010000048.1). Interestingly, these genes have similar expression profiles during infection with these pathogens (Blackman et al., 2015; Engelbrecht et al., 2021). This observation suggests the potential significance of these regions for *Phytophthora* infection. Future research could investigate these genomic regions across various *Phytophthora* spp. to elucidate their evolutionary history. RXLRs effectors are key pathogenicity and virulence factors in oomycetes, known for their diverse functions, including suppression of host defenses. In contrast with our findings on CAZymes repertoires in *P. cinnamomi*, < 50% of RXLRs shared orthology among the *P. cinnamomi* isolates investigated. Moreover, < 30% of the Pc2113 RXLRs DE after infection shared orthology with all *Phytophthora* spp. (OG7457), suggesting isolate- and host-specific roles for RXLR effectors during *P. cinnamomic*–plant interactions. Phylogenetic analysis revealed a subclade exclusive to Pc2113 and Pc2109, which display an expansion of tandem-duplicated RXLRs in each isolate. Interestingly, none of the RXLRs within this subclade are expressed in any of the Pc2113 RNA-seq data, as these genes do not contain transcript count data (*data not shown*). Furthermore, 66.7% of these tandem-duplicated RXLRs in Pc2109 are in GSRs, while none of the Pc2113 RXLRs are in GSRs, suggesting that the more virulent isolate, Pc2109, has expanded its RXLR repertoire through duplication events within plastic regions of the genome (GSRs) to further enhance its virulence. Further experiments will test the Pc2109 expression profile of these expanded RXLRs after pathogen infection. Comparable to the expression profiles of other effectors in this study, more RXLRs were DE in avocado, followed by *N. benthamiana* and *A. thaliana*. A small number of RXLRs were DE in all hosts, suggesting an important role during *P. cinnamomi* infections, such as suppression of plant immunity. Consistent with this hypothesis, one of these commonly upregulated RXLRs was a homolog of *P. sojae* Avr1b and resided in GSRs, suggesting an important role during *P. cinnamomi* infection process. Overexpression of Avr1b in *P. sojae* enhanced its virulence by targeting the E3 ubiquitin ligase PUB1 to manipulate the ubiquitin-mediated degradation pathway (Shan et al., 2004; Dou et al., 2008; Duplan and Rivas, 2014; Lin et al., 2021). The possibility that this Pc2113 Avr1b homolog also targets E3 ubiquitin ligases to enhance virulence needs to be elucidated. In this study, the number of Pc2113 RXLRs DE at the zoospore stage was consistent with the number of RXLRs previously reported in *P. cinnamomi* pre-infection structures (Reitmann et al., 2017). The number and expression profiles of DE Pc2113 RXLRs during host infection via detached leaf inoculations aligned with those reported for GKB4 in avocado root infections (Engelbrecht et al., 2021; Joubert et al., 2021). For example, Pc2113RXLR057, Pc2113RXLR062, and Pc2113RXLR107 clustered with GKB4 RXLRs QVE55538.1, QVE55565.1, QVE55566.1, QVE55567.1, QVE55568.1, and QVE55569.1 exhibited similar upregulation patterns in avocado and were also DE in *N. benthamiana* and *A. thaliana*, suggesting an important role during infection. Several Pc2113 RXLRs exhibited different expression profiles compared to their GKB4 homologs in infected avocado roots. Notably, Pc2113RXLR135, homologous to GKB4 PcinRxLR10 (NCBI accession number QVE55524.1), exhibited upregulation in avocado leaves, whereas PcinRxLR10 was not DE in avocado roots (Joubert et al., 2021). Similarly, GKB4 PcinRxLR47 (NCBI accession number QVE55553.1) and PcinRxLR38 (NCBI accession number QVE55549.1) were not DE in avocado roots, but their corresponding Pc2113 homologs (Pc2113RXLR065 and Pc2113RXLR124) were significantly upregulated in avocado leaves, with Pc2113RXLR065 also DE in *N. benthamiana* (Figures 7C, E). GKB4 PcinRxLR65 (NCBI accession number QVE55564.1) showed no differential expression in avocado roots, while its Pc2113 homolog (Pc2113RXLR061) was upregulated in all hosts and the zoospore stage (Supplementary Tables S4, S6). These findings highlight the dynamic expression patterns of Pc2113 and GKB4 RXLRs across various hosts, infection stages, and tissue types. Understanding these dynamics, including their commonalities and differences, is crucial for elucidating the pathogenic mechanisms of *P. cinnamomi* to develop effective pathogen control strategies. We also identified several Pc2113 RXLRs that shared orthology with *P. infestans* and *P. sojae* RXLRs. The Pc2113RXLR065, homolog to the *P. infestans* RXLR Avr4 (NCBI accession number ABV66276.1), was upregulated in *N. benthamiana* and avocado. PiAvr4 elicits R4-mediated resistance in potatoes (van Poppel et al., 2008) and was expressed at 12 hpi in detached potato leaves (Yin et al., 2017). Similarly, Pc2113RXLR048 is the homolog of *P. sojae* RXLR Avh241 (NCBI accession number AEK81007.1), which elicits cell death in *N. benthamiana* (Wang et al., 2011) and was expressed during the cyst stage and early infection of soybean leaves (Yu et al., 2012). In agreement, we found that Pc2113RXLR048 was also upregulated in avocado and the zoospore stage. Overall, these results suggest that, contrary to the CWDEs, *P. cinnamomi* RXLR effectors might be more important for host-specific infection strategies than general infection strategies as CWDEs; however, functional studies of these effectors are needed to test this hypothesis.

This study expands the genomic resources for *P. cinnamomi* and uncovers potential mechanisms, such as polyploidization and a two-speed genome architecture, that these isolates use to enhance phenotypic diversity. These adaptations, despite their clonality, enable them to thrive in new environments and overcome the resistance of commercially available host rootstocks used in California, such as Dusa (Belisle et al., 2019b). The DE analyses of Pc2113 infecting different hosts, coupled with orthology analysis, provide valuable insights into the common infection strategies employed by several *Phytophthora* spp. and the common strategies that *P. cinnamomi* employs to cause infection in several hosts and in specific hosts. Moreover, despite the methodological differences, variation in *Phytophthora* spp. analyzed, and host plants and tissue types used in the previously mentioned RNA-seq studies, we were able to identify several effector homologs in Pc2113 that exhibited similar expression patterns to effectors described in those respective studies. Together, our results provide a useful framework for the selection of candidate *P. cinnamomi* effectors for functional validation *in planta* and the identification of common host susceptibility targets to break *Phytophthora*- and *P. cinnamomi* susceptibility to aid in the development of resistant crops. Finally, this study demonstrates the use of detached leaf assays and model plants, such as *A. thaliana* and *N. benthamiana*, to study the molecular *P. cinnamomic*–plant interactions since we found similar and overlapping expression patterns of different effectors previously reported in *P. cinnamomi–*avocado interactions (Engelbrecht et al., 2021; Joubert et al., 2021; Backer et al., 2022) and in other *Phytophthora*–plant pathosystems.

## Data availability statement

The datasets presented in this study can be found in online NCBI repositories using the accession number(s): https://www.ncbi.nlm.nih.gov/, PRJNA1019738; https://www.ncbi.nlm.nih.gov/, PRJNA1019754; https://www.ncbi.nlm.nih.gov/, PRJNA1019764. The code and orthogroup data used in this study can be found in the GitHub repository Pcinnamomi-Genomics: https://github.com/aidanshands/Pcinnamomi-Genomics. The genomes and annotations can be accessed at Dryad (https://doi.org/10.5061/dryad.bcc2fqzkb) for Pc2113 and Dryad (https://doi.org/10.5061/dryad.63xsj3v8g) for Pc2109.

## Author contributions

AS: Writing – review & editing, Writing – original draft, Visualization, Validation, Software, Methodology, Investigation, Formal analysis, Data curation. GX: Writing – review & editing, Methodology, Investigation, Formal analysis. RB: Writing – review & editing, Methodology, Investigation. SS: Writing – review & editing, Methodology, Formal analysis. NJ: Writing – review & editing, Methodology, Formal analysis. AB: Writing – review & editing, Supervision, Methodology, Investigation, Formal analysis. LC: Writing – review & editing, Supervision, Methodology, Investigation. PM: Writing – review & editing, Writing – original draft, Validation, Supervision, Resources, Project administration, Methodology, Investigation, Funding acquisition, Data curation, Conceptualization.
