# Supplementary material for: Genomic and transcriptomic analyses of Phytophthora cinnamomi reveal complex genome architecture, expansion of pathogenicity factors, and host-dependent gene expression profiles
Source: Front Microbiol. 2024 Aug 15;15:1341803. doi: 10.3389/fmicb.2024.1341803 (PMC11357935; doi:10.3389/fmicb.2024.1341803)
Supplement: Supplementary file 1 [file Data_Sheet_1.DOCX]

Supplementary Material

Genomic and transcriptomic analyses of *Phytophthora cinnamomi* reveals complex genome architecture, expansion of pathogenicity factors, and host-dependent gene expression profiles

**Aidan C. Shands^1^, Guangyuan Xu^1^, Rodger J. Belisle^1^, Shirin Seifbargui^1^, Natasha Jackson^1^, Aureliano Bombarely^2^, Liliana M. Cano^3^, and Patricia M. Manosalva^1*^**

^1^Department of Microbiology and Plant Pathology, University of California Riverside, Riverside, CA, United States.

^2^Instituto de Biología Molecular y Celular de Plantas, Consejo Superior de Investigaciones Científicas-Universidad Politécnica de Valéncia, Valencia, Spain.

^3^Department of Plant Pathology, Institute of Food and Agricultural Sciences IFAS, Indian River Research and Education Center IRREC, University of Florida, Fort Pierce, FL, United States.

*** Correspondence:**Patricia M. Manosalva
[Patricia.manosalva@ucr.edu](mailto:Patricia.manosalva@ucr.edu)

**Supplementary Methods**

**Supplementary Method 1. Delimitating the genomes of Pc2113 and Pc2109**

First, we simulated single-copy ‘core’ orthologs (n=2540) content determined by Orthofinder in GDRs and GSRs as percent of total genes belonging to each of these regions using values of the length ‘L’ of the FIRS between genes ranging from 100 bp to 5 Kb with 100bp increments. Genes with both FIRs greater than L were considered GSR genes and genes with both FIRs below L were considered GDR genes. Genes that had one FIR larger than L and the other lower than L were considered in-between, and genes with one FIR missing was considered not determined (ND). The core ortholog segregation rate was defined as the difference between the core ortholog content within GDRs and GSRs, respectively. To determine the optimal L value that best fits the data, and that maximized the segregation rate and in which the percentage of core ortholog genes residing in GDR or in-between corresponded to at least 90%.

**Supplementary Method 2. Predicting RXLR effector proteins**

The regular expressions used are as follows: RXLR (r"^[a-yA-Y]{10,110}?R[A-Y]LR"); EER (r"^[a-yA-Y]{34,150}?EER"); QXLR (r"^[a-yA-Y]{10,110}?Q[A-Y]LR"); RXL (r"^[a-yA-Y]{10,110}?R[A-Y]L[^R]"); XLR (r"^[a-yA-Y]{10,110}?[^QR][A-Y]LR"); QXLR-EER (r"^[a-yA-Y]{10,110}Q[A-Y]LR[A-Y]*EER"); RXL-EER (r"^[a-yA-Y]{10,110}R[A-Y]L[A-Y]*EER"); RXLR-EER (r"^[a-yA-Y]{10,110}R[A-Y]LR[A-Y]*EER"); XLR-EER (r"^[a-yA-Y]{10,110}[A-Y][A-Y]LR[A-Y]*EER"). The candidate RXLR effectors were selected based on the following criteria: (1) the RXLR position must be between 30 and 60 amino acids; (2) the RXLR position must be downstream of the signal peptide cleavage site (Win et al., 2007). The candidates were combined into a larger dataset and redundant candidates were removed. The candidate RXLR effectors were subjected to a WY-domain search with sequences described in Boutemy et al. (2011) using HMMER v3.2.1 (Eddy, 2009) and were considered to have a putative WY-domain if the e-value < 0.05. Putative RXLRs were removed if they (1) have been identified as a CAZYme, (2) identified as an apoplastic effector as determined by EffectorP, and (3) if the protein was not predicted by EffectorP, only had one predicted motif (REGEX) and had a functional annotation that was unrelated. The final putative RXLR dataset was used for all analyses described in this paper.
