# Supplementary material for: Genomic and transcriptomic analyses of Phytophthora cinnamomi reveal complex genome architecture, expansion of pathogenicity factors, and host-dependent gene expression profiles
Source: Front Microbiol. 2024 Aug 15;15:1341803. doi: 10.3389/fmicb.2024.1341803 (PMC11357935; doi:10.3389/fmicb.2024.1341803)
Supplement: Supplementary file 2 [file Table_1.DOCX]

Supplementary Material

**Supplementary Table 1.** Oligonucleotides used in this study to validate the effector gene expression after *P. cinnamomi* infection in *N. benthamiana* using qRT-PCR.

| **Primer Name** | **Primer Sequence** | **Construct** |
| --- | --- | --- |
| 2113_PC00002279g0000010_F_64bp | ACCACTGACGCTGAGCTGAC | qPCR Forward primer for gene expression analyses |
| 2113_PC00002279g0000010_R_299bp | TCCAACCTGCCCAGTAGCTT | qPCR Reverse primer for gene expression analyses |
| 2113_PC00002373g0000080_F_110bp | CGGACCAGTACATCGACCAA | qPCR Forward primer for gene expression analyses |
| 2113_PC00002373g0000080_R_444bp | CGTTGTCGCAGTAGGAGCTG | qPCR Reverse primer for gene expression analyses |
| 2113_PC00002266g0000100_F_426bp | TATCAAGAAGCAGGCGGACA | qPCR Forward primer for gene expression analyses |
| 22113_PC00002266g0000100_R_730bp | GCACCTTCTGGACCCACTTC | qPCR Reverse primer for gene expression analyses |
| Pc_ Ws21 (86101) _F | CTGTTCTGCATCGCCTTCAC | qPCR Forward primer for reference gene |
| Pc_ Ws21 (86101) _R | GCGGATGTACACGTTCTGGA | qPCR Reverse primer for reference gene |
