# Supplementary material for: Genomic and transcriptomic analyses of Phytophthora cinnamomi reveal complex genome architecture, expansion of pathogenicity factors, and host-dependent gene expression profiles
Source: Front Microbiol. 2024 Aug 15;15:1341803. doi: 10.3389/fmicb.2024.1341803 (PMC11357935; doi:10.3389/fmicb.2024.1341803)
Supplement: Supplementary file 3 [file Table_2.DOCX]

***Supplementary Material***

**Supplementary Table 2.** Summary of the DNA sequencing data used in this study.

| **Isolate** | **Species** | **Sequencing Platform** | **Service** | **Number of raw bases (Gb)** | **Number of filtered bases (Gb)** | **Number of filtered reads** | **Average filtered read length (bp)** |
| --- | --- | --- | --- | --- | --- | --- | --- |
| Pc2113 | *P. cinnamomi* | PacBio Sequel | Novogene | 14.97^a^ | 14.95^b^ | 1,164,148^b^ | 12,846^b^ |
|  |  | PacBio | Mount Sinai | 5.91^a^ | 5.04^b^ | 643,297^b^ | 7,829^b^ |
|  |  | Illumina Novaseq 6000 | Novogene | 24.64 | 23.07^c^  18.9^d^ | 156,953,904^c^ 125,821,562^d^ | 147^c,d^ |
| Pc2109 | *P. cinnamomi* | PacBio Sequel | Novogene | 12.04^a^ | 12.03^b^ | 1,139,687^b^ | 10,555^b^ |
|  |  | PacBio | Mount Sinai | 5.43^a^ | 4.9^b^ | 631,504^b^ | 7,757^b^ |
|  |  | Illumina Novaseq 6000 | Novogene | 20.14 | 19.11^c^  14.4^d^ | 130,010,164^c^ 95,964,962^d^ | 147^c^  149^d^ |
| CBS 144.22 | *P. cinnamomi* | Illumina Novaseq 6000 | Novogene | 9.10 | 6.2^d^ | 41442502^d^ | 149^d^ |
| Pi1306-C | *P. infestans* | Illumina MiSeq | Pan et al., (2018) | 12.70 | 6.3^d^ | 28709418^d^ | 219^d^ |

^a^ Indicates PacBio raw polymerase reads. ^b^ Indicates PacBio subreads. ^c^ Indicates reads filtered with trimmomatic. ^d^ Indicates reads filtered with fastq-mcf.
