# Supplementary material for: Genomic and transcriptomic analyses of Phytophthora cinnamomi reveal complex genome architecture, expansion of pathogenicity factors, and host-dependent gene expression profiles
Source: Front Microbiol. 2024 Aug 15;15:1341803. doi: 10.3389/fmicb.2024.1341803 (PMC11357935; doi:10.3389/fmicb.2024.1341803)
Supplement: Supplementary file 9 [file Table_8.DOCX]

Supplementary Material

**Supplementary Table 8.** Summary of the differentially expressed genes (DEGs) between each host and timepoint. All genes in this table have a |LFC| ≥ 2 and an adjusted P-value < 0.05. Up refers to up-regulated genes and down refers to down-regulated genes.

| **Host** | **16 HPI** | | | **24 HPI** | | | **90 HPI** | | |
| --- | --- | --- | --- | --- | --- | --- | --- | --- | --- |
|  | **DEGs** | **Up** | **Down** | **DEGs** | **Up** | **Down** | **DEGs** | **Up** | **Down** |
| *A. thaliana* | 249 | 241 | 8 | 288 | 267 | 21 | 562 | 238 | 324 |
| *N. benthamiana* | 1,820 | 26 | 1,794 | 3,121 | 1,162 | 1,881 | NA | NA | NA |
| *Avocado* | 5,113 | 1,877 | 2,857 | 5,678 | 1,844 | 3,320 | NA | NA | NA |
