# Supplementary figures and images for: Genomic and transcriptomic analyses of Phytophthora cinnamomi reveal complex genome architecture, expansion of pathogenicity factors, and host-dependent gene expression profiles
Source: Front Microbiol. 2024 Aug 15;15:1341803. doi: 10.3389/fmicb.2024.1341803 (PMC11357935; doi:10.3389/fmicb.2024.1341803)

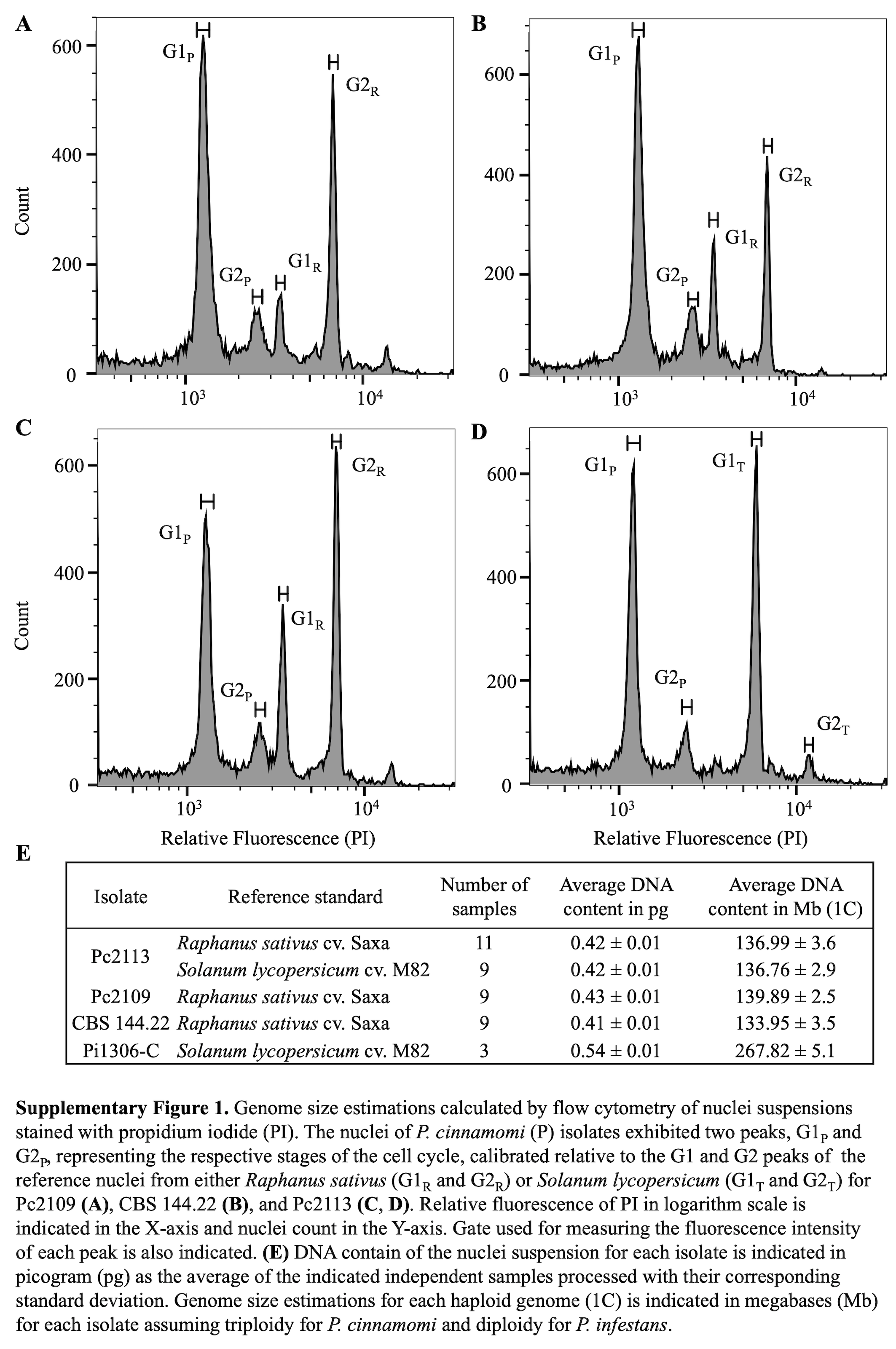

Supplement: Supplementary file 11 [file Image_1.TIFF]

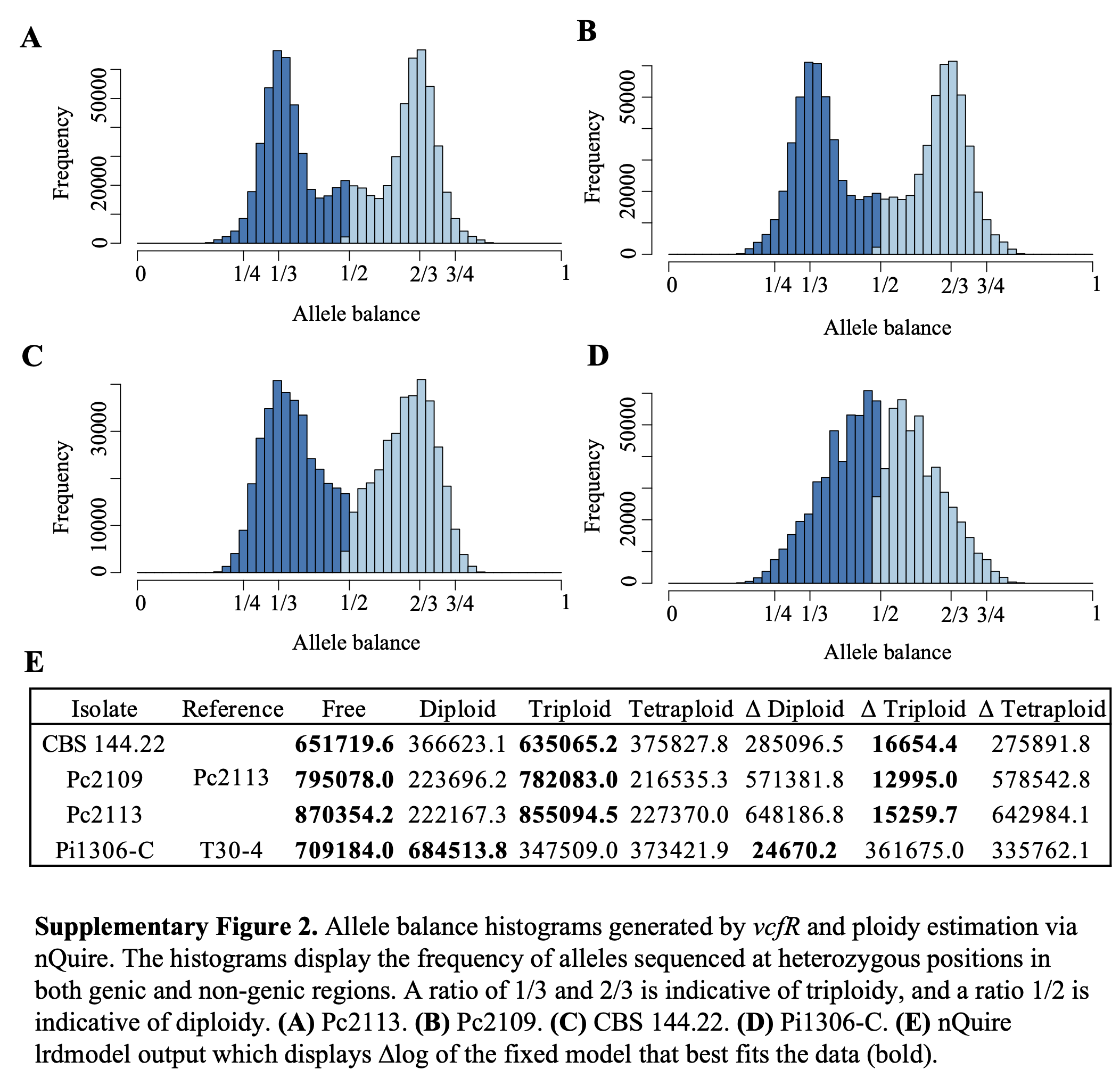

Supplement: Supplementary file 12 [file Image_2.TIFF]

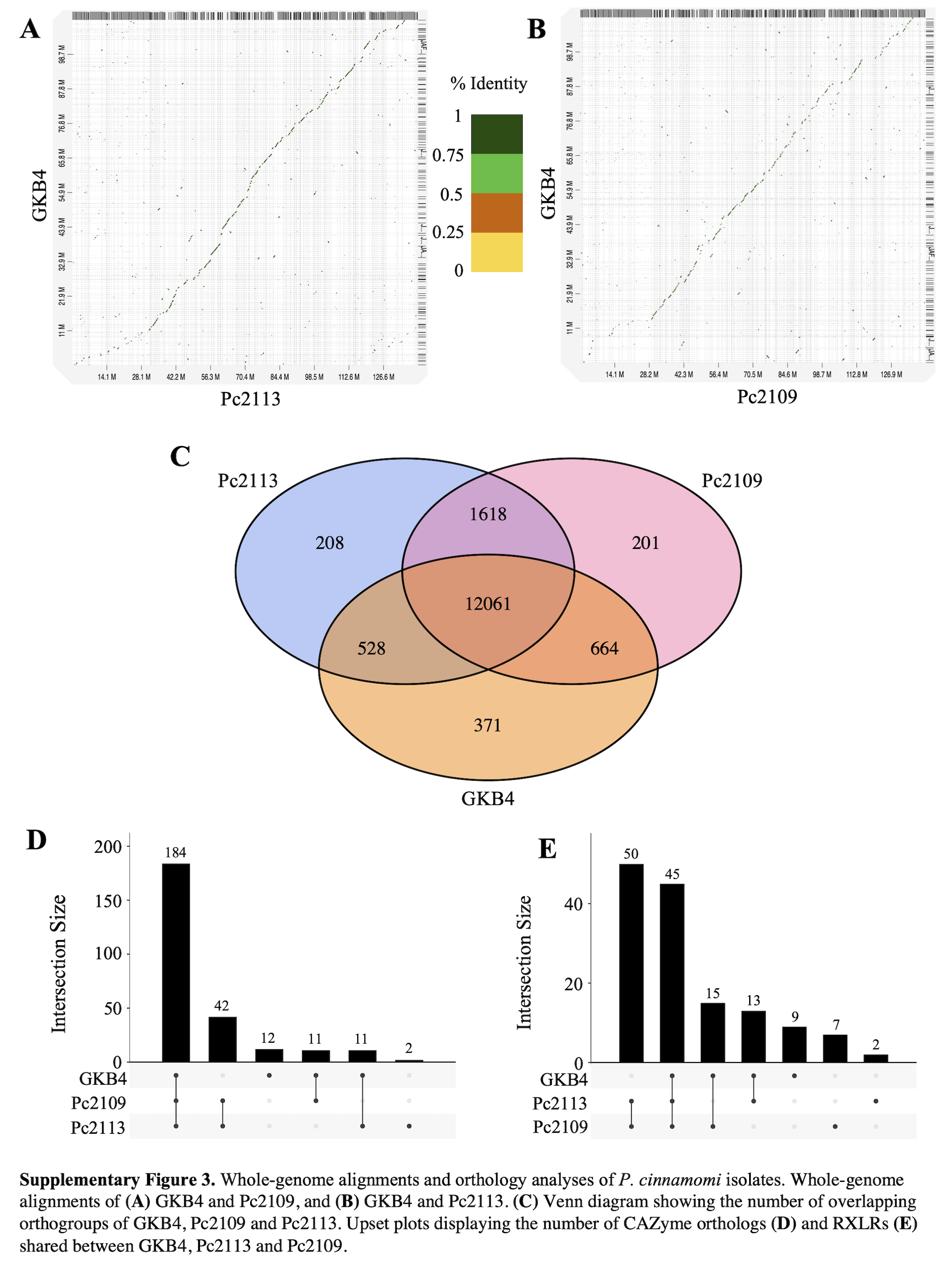

Supplement: Supplementary file 13 [file Image_3.TIFF]

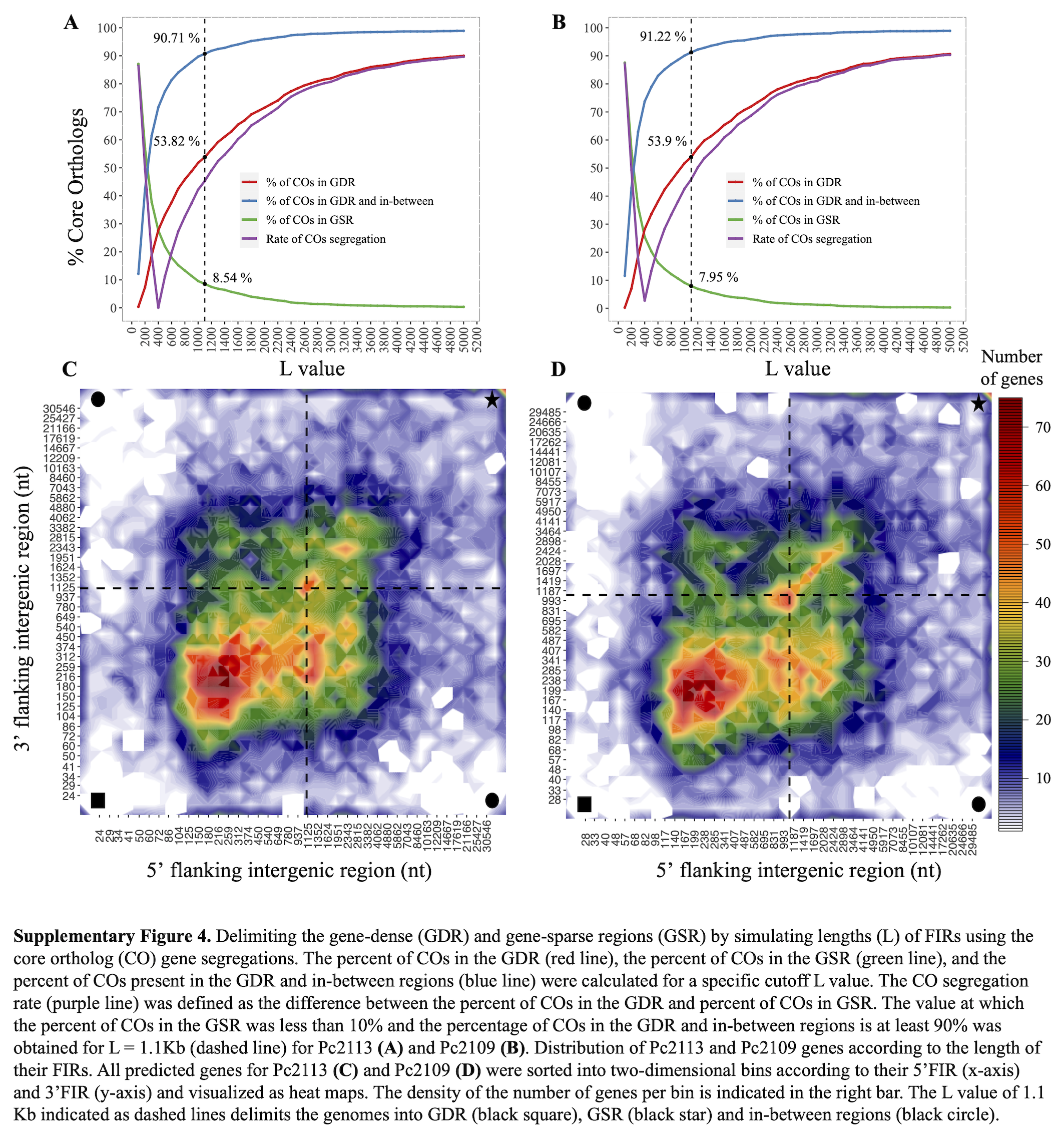

Supplement: Supplementary file 14 [file Image_4.TIFF]
